# *Staphylococcus aureus* uses the ArlRS and MgrA cascade to regulate immune evasion during skin infection

**DOI:** 10.1016/j.celrep.2021.109462

**Published:** 2021-07-27

**Authors:** Jakub M. Kwiecinski, Rachel M. Kratofil, Corey P. Parlet, Bas G.J. Surewaard, Paul Kubes, Alexander R. Horswill

**Affiliations:** 1 Department of Immunology and Microbiology, University of Colorado School of Medicine, Aurora, CO 80045, USA; 2 Department of Microbiology, Faculty of Biochemistry, Biophysics and Biotechnology, Jagiellonian University, Krakow 30387, Poland; 3 Department of Physiology and Pharmacology, University of Calgary, Calgary, AB T2N 4Z6, Canada; 4 Calvin, Phoebe, and Joan Snyder Institute for Chronic Diseases, University of Calgary, Calgary, AB T2N 4Z6, Canada; 5 Department of Veterans Affairs, Iowa City VA Medical Center, Iowa City, IA 52246, USA; 6 Department of Microbiology and Immunology, University of Iowa, Iowa City, IA 52242, USA; 7 Department of Veterans Affairs, Eastern Colorado Health Care System, Aurora, CO 80045, USA; 8 Lead contact

## Abstract

Skin is one of the most common sites of host immune response against *Staphylococcus aureus* infection. Here, through a combination of *in vitro* assays, mouse models, and intravital imaging, we find that *S. aureus* immune evasion in skin is controlled by a cascade composed of the ArlRS two-component regulatory system and its downstream effector, MgrA. *S. aureus* lacking either ArlRS or MgrA is less virulent and unable to form correct abscess structure due to de-repression of a giant surface protein, Ebh. These *S. aureus* mutants also have decreased expression of immune evasion factors (leukocidins, chemotaxis-inhibitory protein of *S. aureus* [CHIPS], staphylococcal complement inhibitor [SCIN], and nuclease) and are unable to kill neutrophils, block their chemotaxis, degrade neutrophil extracellular traps, and survive direct neutrophil attack. The combination of disrupted abscess structure and reduced immune evasion factors makes *S. aureus* susceptible to host defenses. ArlRS and MgrA are therefore the main regulators of *S. aureus* immune evasion and promising treatment targets.

## In brief

Kwiecinski et al. show that *Staphylococcus aureus* uses the ArlRS-MgrA regulatory system to coordinate gene expression during skin infection. This cascade is required for proper abscess structuring and evasion of the host innate immune system, which together are essential for full *S. aureus* virulence.

## Graphical Abstract

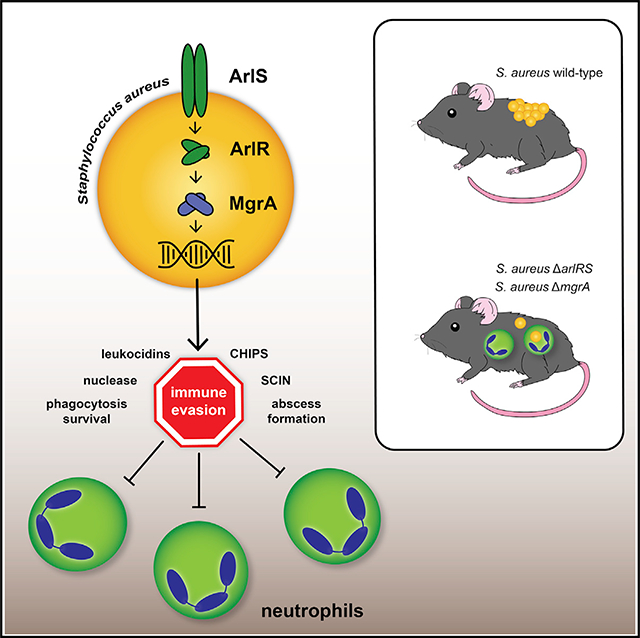

## INTRODUCTION

Skin with its underlying tissues is often the first line of defense against pathogens, both as a physical barrier and as the site of the initial immune response. *Staphylococcus aureus* is the leading cause of the skin and soft tissue infections, with up to 80% of cases attributable to this pathogen (Ray et al., 2013). Skin infections are also the most common type of staphylococcal disease (Jacobsson et al., 2007; Landrum et al., 2012). While *S. aureus* skin infections usually remain self-limiting, they become recurrent in ~20% of patients (Sreeramoju et al., 2011). They can also lead to systemic spread if the immune system fails to contain the pathogen, which makes skin and soft tissue infections the main source of the *S. aureus* bacteremia (Wilson et al., 2011). Recently, treatment of staphylococcal skin infections has been increasingly challenging, and associated morbidity, mortality, and healthcare costs have been rising, partly due to growing prevalence of methicillin-resistant *S. aureus* (MRSA) (Bassetti et al., 2017). In order to design better treatments against *S. aureus* skin infections, an improved understanding is needed of the mechanisms for establishing infectious foci and evading the local immune response.

Neutrophils are the first immune cells recruited to the site of skin invasion by *S. aureus* and are essential for clearing *S. aureus* from the tissue and preventing its systemic spread (Kwiecinski et al., 2014; Mölne et al., 2000). Neutrophils kill the invading pathogen through phagocytosis, production of reactive oxygen species (ROS) and antimicrobial peptides, and trapping bacteria in neutrophil extracellular traps (NETs). At the same time, neutrophils actively create the structure of skin abscesses (Brandt et al., 2018; Kobayashi et al., 2015). In order to mount an effective response to these immune attacks and adapt to the novel skin niche, *S. aureus* must orchestrate a precise and timely production of a number of virulence factors (Balasubramanian et al., 2017). To achieve such coordination, *S. aureus* relies on dedicated regulatory systems (Haag and Bagnoli, 2017; Jenul and Horswill, 2019), such as the Agr quorum-sensing system, the SaeRS two-component system, and the CodY nutritional regulator, all of which have been shown to control virulence during skin infections (Cheung et al., 2011; Kobayashi et al., 2011; Montgomery et al., 2010, 2012; Nygaard et al., 2010). However, many other important regulatory systems have not been investigated in this context, including the ArlRS-MgrA regulatory cascade, a system essential for *S. aureus* virulence in the bloodstream and in other systemic infections (Chen et al., 2009; Crosby et al., 2016b; Gupta et al., 2013; Jonsson et al., 2008; Li et al., 2019; Liu et al., 2014; Radin et al., 2016; Walker et al., 2013). This cascade begins with the ArlRS two-component regulator, which is composed of the membrane-bound sensory kinase ArlS and the response regulator ArlR. After sensing a yet-unknown signal, ArlS phosphorylates ArlR, thus making this DNA-binding regulator active. The activated ArlR in turn drives expression of the regulators MgrA and Spx. The Spx regulator controls *S. aureus* response to beta-lactam antibiotics and stress, while the global regulator MgrA directly impacts virulence by controlling expression of over 100 effector genes (Bai et al., 2019; Crosby et al., 2020). The importance of this ArlRS and MgrA cascade in systemic bloodstream infections made us suspect that it might play an essential role also in the context of a localized skin infection.

In this work, we identified the ArlRS and MgrA as regulators of the virulence in MRSA skin infection, affecting both skin damage and MRSA survival in the host. These effects were largely due to ArlRS and MgrA regulating MRSA immune evasion through control of virulence factor expression. Immune evasion was also impacted by the altered spatial organization of the abscess where MRSA is shielded from host phagocytes.

## RESULTS

### ArlRS and MgrA regulate virulence in skin infection

**Figure 1.**
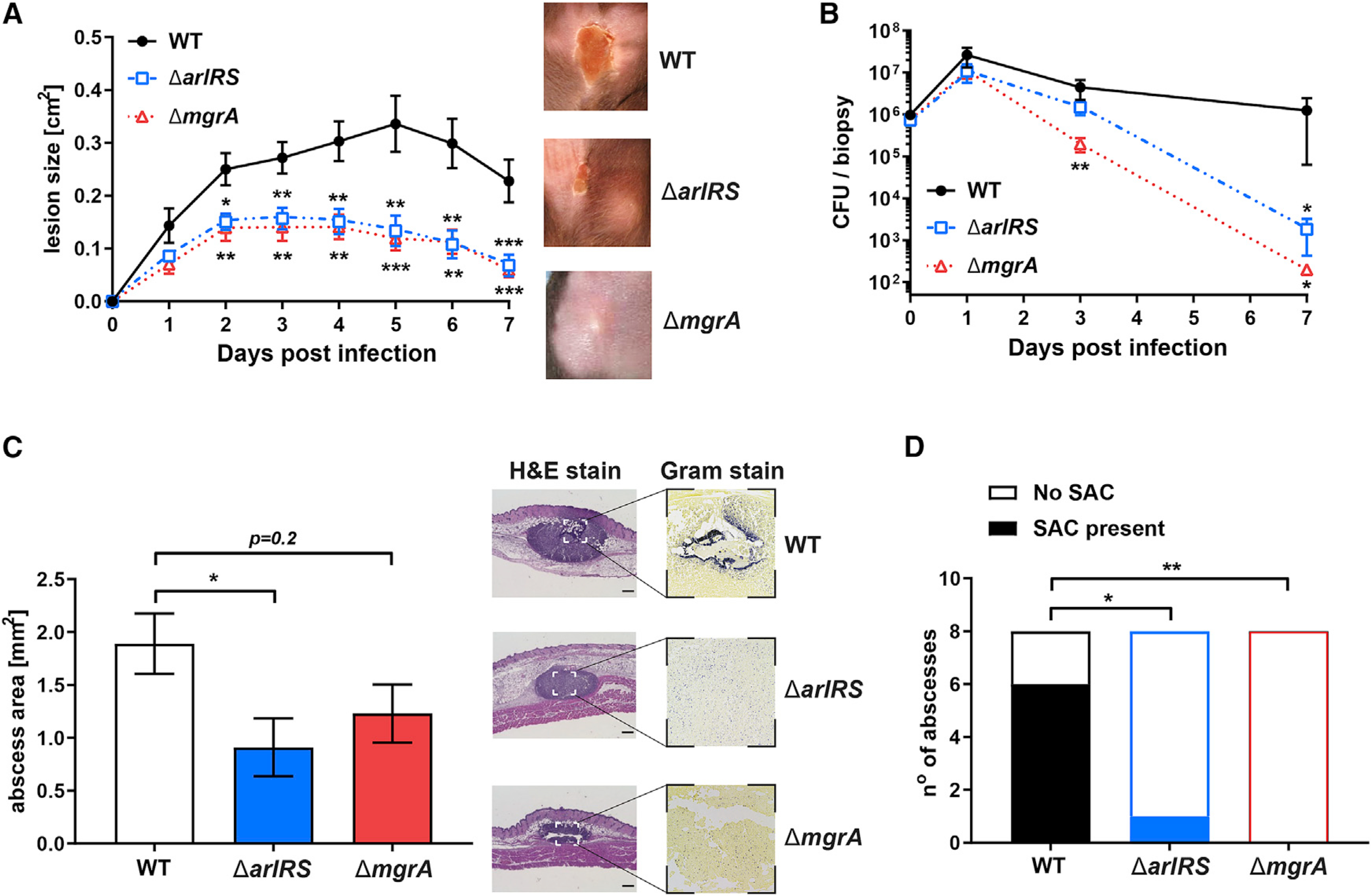
ArlRS and MgrA control *S. aureus* skin infection severity (A) C57BL/6 mice were infected with *S. aureus* through subcutaneous injection, and developing skin infection was observed. The size of dermonecrotic lesions was measured daily. (B) On selected days, the infected areas were biopsied, and skin bacterial burden in homogenized biopsy specimens was measured. (C and D) Additionally, skin biopsy specimens were taken on day 1 of infection, and histopathological sections of the biopsy specimens were used to measure the size of abscesses formed in skin (C) and the presence of tightly clumped staphylococcal abscess communities (SACs) inside these abscesses (D). Scale bars, 300 μm. Data are shown as mean ± SEM. N = 9 (A), 5–8 (B), and 8 (C and D). *p < 0.05; **p < 0.01; ***p < 0.001. All p values are for comparisons to WT.

When injected subcutaneously into mice, the wild-type (WT) USA300 MRSA strain LAC (hereafter referred to as MRSA WT) caused a pronounced skin infection, associated with a gradual development of skin necrosis. This virulent process was significantly attenuated when mutants lacking elements of the ArlRS regulatory cascade, either deletions of Δ*arlRS* or Δ*mgrA*, were injected. In mutants, necrotic lesions were developing slower and never reached the extent observed in the MRSA WT (Figure 1A). Decreased virulence of the ArlRS and MgrA mutants was also evident when lower dose of MRSA was injected, leading to skin abscess formation rather than to the immediate skin necrosis. In this setting, all strains managed to proliferate to a similar extent during the first day of infection (with a trend toward fewer colony-forming units [CFUs] in mutants). Afterward, the MRSA WT persisted in skin, while CFUs of the Δ*arlRS* and Δ*mgrA* mutants decreased significantly (Figure 1B). This accelerated clearance of the mutant strains by the host suggests a defect in their immune evasion mechanisms. The histopathological examination of skin showed that the Δ*arlRS* and Δ*mgrA* mutants formed slightly smaller abscesses (Figure 1C), but the most striking differences occurred in bacterial organization within the abscesses, and this was especially noticeable in Gram stains of the tissue (Figure 1C). MRSA WT formed typical abscess structure, with a densely packed bacteria in the center of the abscess, the so-called staphylococcal abscess community (SAC) (Cheng et al., 2011). The Δ*arlRS* and Δ*mgrA* mutants failed to form this type of tightly clumped community and were present dispersed across the abscess (Figure 1D). SACs are thought to protect *S. aureus* from host phagocytes (Cheng et al., 2011); therefore, this improper spatial organization of the cascade mutants could contribute to the accelerated clearance and the reduced virulence of mutant strains.

### ArlRS-MgrA cascade regulates adhesion of *S. aureus* to skin cells

During infection, *S. aureus* adheres to and invades host cells, finding inside them a shelter from host phagocytes (Fraunholz and Sinha, 2012; Sinha et al., 1999). When adhesion to dermal fibroblasts was tested, the Δ*arlRS* and Δ*mgrA* mutants showed an adhesion defect compared to MRSA WT (Figure S1A). This adhesion deficit disappeared after chromosomal complementation of the missing genes (Figure S1A). The Δ*arlRS* and Δ*mgrA* mutants are known to de-repress the expression of the large surface proteins Ebh (extracellular-matrix-binding protein homolog) and SraP (serine-rich adhesin to platelets), which interfere with proper binding of *S. aureus* to host endothelial cells (Kwiecinski et al., 2019). Therefore, possible role of de-repressed Ebh and SraP in the decreased attachment to skin fibroblast was tested. As predicted, normal adhesion to skin fibroblasts was partially or fully restored when genes for SraP and Ebh were deleted in the Δ*arlRS* and Δ*mgrA* backgrounds (Figures S1B and S1C), showing that de-repression of these large proteins is responsible for the observed defect of fibroblast attachment in the cascade mutant strains. It is, however, unlikely that altered interaction with cells in dermis would alone explain either the altered spatial organization or the accelerated immune clearance of the mutant strains.

### ArlRS and MgrA regulate spatial organization of *S. aureus* model *in vitro* abscesses

**Figure 2.**
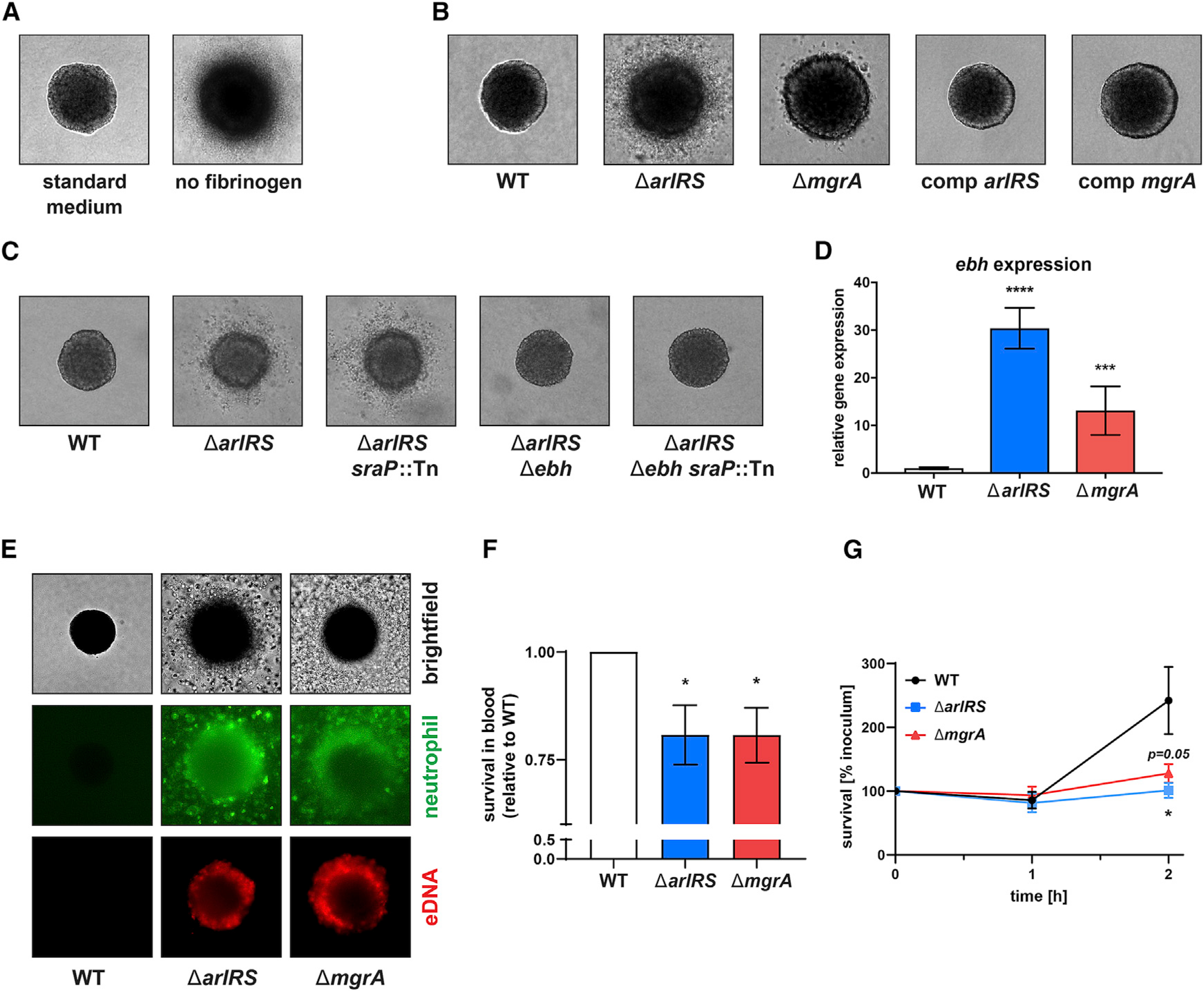
ArlRS and MgrA control formation and immune evasion of model *in vitro S. aureus* abscess communities (A–C) Three-dimensional SACs formed from individual *S. aureus* cells after culturing in collagen/fibrinogen/RPMI gels for 16 h. These were used to determine the role of fibrinogen present in the culture medium (A), the effects of mutations in the ArlRS-MgrA signaling system (B), and the role of giant surface proteins SraP and Ebh in causing the starburst phenotype in the Δ*arlRS* mutant strains (C). (D) Expression of *ebh* in mid-exponential *S. aureus* RPMI culture was measured with qPCR and normalized to *gyrB* expression. (E) Behavior of human neutrophils (stained green with CFDA-SE) 3 h after addition to the *in vitro* three-dimensional abscess models was also visualized, with propidium iodide (PI) added before imaging to stain extracellular DNA and lysed cells. (F) Survival of *S. aureus* after 1-h incubation with fresh human blood was quantified and normalized to WT survival. (G) Survival of *S. aureus* co-incubated with purified human neutrophils was measured. Representative images are shown. Image size: 350 × 350 μm. Data are shown as mean ± SEM. n = 6 (D and F) or 5 (G). *p < 0.05; ***p < 0.001; ****p < 0.0001. All p values are for comparisons to WT. See also Figures S2–S4.

To understand the mechanism behind the altered spatial organization inside skin abscesses, we used a three-dimensional model of MRSA growing inside collagen gels in a presence of fibrinogen, which replicates properties of the SAC (Guggenberger et al., 2012). While growing in this dermis-like matrix, MRSA WT formed tightly clumped spherical SAC-like communities (Figure 2A). This process required presence of the fibrinogen; when fibrinogen was not added to the medium, MRSA failed to form a typical tight community and displayed instead a “starburst”-like phenotype of individual loose bacteria detaching from the central community (Figure 2A). Moreover, we could demonstrate that formation of SAC-like community requires bacteria to bind fibrinogen from the media on their surface. As *S. aureus* expresses a vast repertoire of different fibrinogen-binding proteins (Crosby et al., 2016a), the importance of fibrinogen binding in the *in vitro* abscess model was tested using *Lactococcus lactis*, since this bacterium lacks fibrinogen-binding proteins of its own. Only simultaneous expression of a prototypical *S. aureus* fibrinogen-binding protein ClfA on *L. lactis* surface, and the presence of fibrinogen in the medium, allowed this bacterium to form tight three-dimensional communities (Figure S2A). This emphasizes the need to bind fibrinogen on bacterial surface in order to properly form a SAC-like abscess structure.

In this *in vitro* abscess model, the Δ*arlRS* and Δ*mgrA* mutants failed to form a correct SAC-like structure and instead showed a starburst phenotype (Figure 2B) that mirrored the WT phenotype without fibrinogen in this assay (Figure 2A). Chromosomal complementation of the *arlRS* and *mgrA* genes restored the abscess phenotype to the WT appearance (Figure 2B). The observed defect of the Δ*arlRS* and Δ*mgrA* mutants paralleled the *in vivo* failure to form SAC in the abscess by the mutants in the mouse infection model (Figure 1D).

Previous work with this *in vitro* model identified Agr quorum-sensing system activation and staphylokinase (Sak) secretion as factors causing spread of bacteria from the abscess community (Guggenberger et al., 2012), but the observed “starburst” phenotype of the Δ*arlRS* and Δ*mgrA* mutants was independent from Agr and Sak. The phenotype occurred even in the strains with Δ*agr* or Δ*sak* backgrounds (Figure S2B). The giant surface proteins SraP and Ebh, upregulated in the absence of ArlRS or MgrA, were previously found to prevent *S. aureus* binding to fibrinogen and to block fibrinogen-induced clumping (Crosby et al., 2016b; Kwiecinski et al., 2019; Walker et al., 2013), making them possible mediators of the starburst phenotype. When tested, lack of SraP in Δ*arlRS* had no impact on the phenotype, but in the absence of Ebh, the Δ*arlRS* Δ*ebh* mutant regained the WT phenotype (Figure 2C). The same pattern of the starburst phenotype being caused by Ebh (despite the phenotype being overall less pronounced) was observed in the Δ*mgrA* background (Figure S2C). Moreover, the elevated expression of *ebh*, but not *sraP*, correlated with the degree of the phenotype in the Δ*arlRS* and Δ*mgrA* mutants (Figures 2D and S2D). This further stresses the role of the Ebh for the starburst phenotype.

As different *S. aureus* strains often have different repertoire of surface proteins, the effect of Δ*arlRS* mutation on formation of model abscesses was tested in divergent *S. aureus* strains. Failure to form the proper three-dimensional SAC in Δ*arlRS* and Δ*mgrA* mutants was seen in all strains that harbor the full-length *ebh* in the genome (strains 502A and MW2), but it did not occur in strains that express only a truncated, nonfunctional version of Ebh (strains N315 and MN8; Figure S3). Altogether, this model suggests that the overexpression of the giant surface protein Ebh, which interferes with bacterial binding of the host fibrinogen, prevents the ArlRS and MgrA mutants from forming the tightly packed SAC and thus from forming the correct spatially organized *S. aureus* abscesses.

### ArlRS and MgrA regulate immune evasion and survival of *S. aureus* interacting with neutrophils

The failure to form tight SAC exposes the Δ*arlRS* and Δ*mgrA* mutants to immune attacks by the host neutrophils infiltrating the infected skin. However, *S. aureus* produces a vast array of immune evasion factors blocking neutrophil functions or directly killing them, which should allow *S. aureus* to escape host phagocytes even in the absence of protective abscess structures. It is therefore possible that altered regulation of such factors additionally accelerated clearance of the mutant strains during infection. When purified human neutrophils were added to *in vitro* abscess models, only a few neutrophils entered into the gel and could be observed in the vicinity of the model SAC of MRSA WT (Figure 2E). Focusing the field of view on the layer immediately above the gel revealed that neutrophils remained on the surface of the gel containing MRSA WT, many of them dead, lysed, or possibly attempting to produce NETs, as demonstrated by propidium iodide staining (Figure S4). In contrast, in the Δ*arlRS* and Δ*mgrA* mutants, neutrophils readily entered the gel, approached the model abscesses, and even penetrated the SAC, directly engaging bacteria (Figure 2E). In case of the mutant strains, staining for extracellular DNA demonstrated bright spots corresponding to individual neutrophils (presumably dead, undergoing lysis, or NETosis), as well as a large amount of diffuse staining suggestive of NETs appearing around the periphery of the SAC and ensnaring the whole community (Figure 2E).

Similarly, when different strains were added to human blood *in vitro* and their survival measured, the Δ*arlRS* and Δ*mgrA* mutants were more susceptible than MRSA WT to killing by blood phagocytes (Figure 2F), though this effect in suspension was not as pronounced as in the model abscesses, possibly due to additional phagocyte-independent killing mechanism present in blood. Notably, when mixed directly with isolated human neutrophils, the MRSA WT strain could evade them and began to proliferate, while the Δ*arlRS* and Δ*mgrA* mutants were kept under control by neutrophils for up to 2 h (Figure 2G). Altogether, the mutant strains lacking the ArlRS-MgrA cascade demonstrate an immune evasion defect, partly related to failure to form the protective three-dimensional structures in the infected site but apparently also due to some other mechanisms, such as possible failure to block phagocyte recruitment and/or phagocytosis and/or killing inside the phagocytes.

### ArlRS and MgrA regulate expression of multiple *S. aureus* immune evasion genes

**Figure 3.**
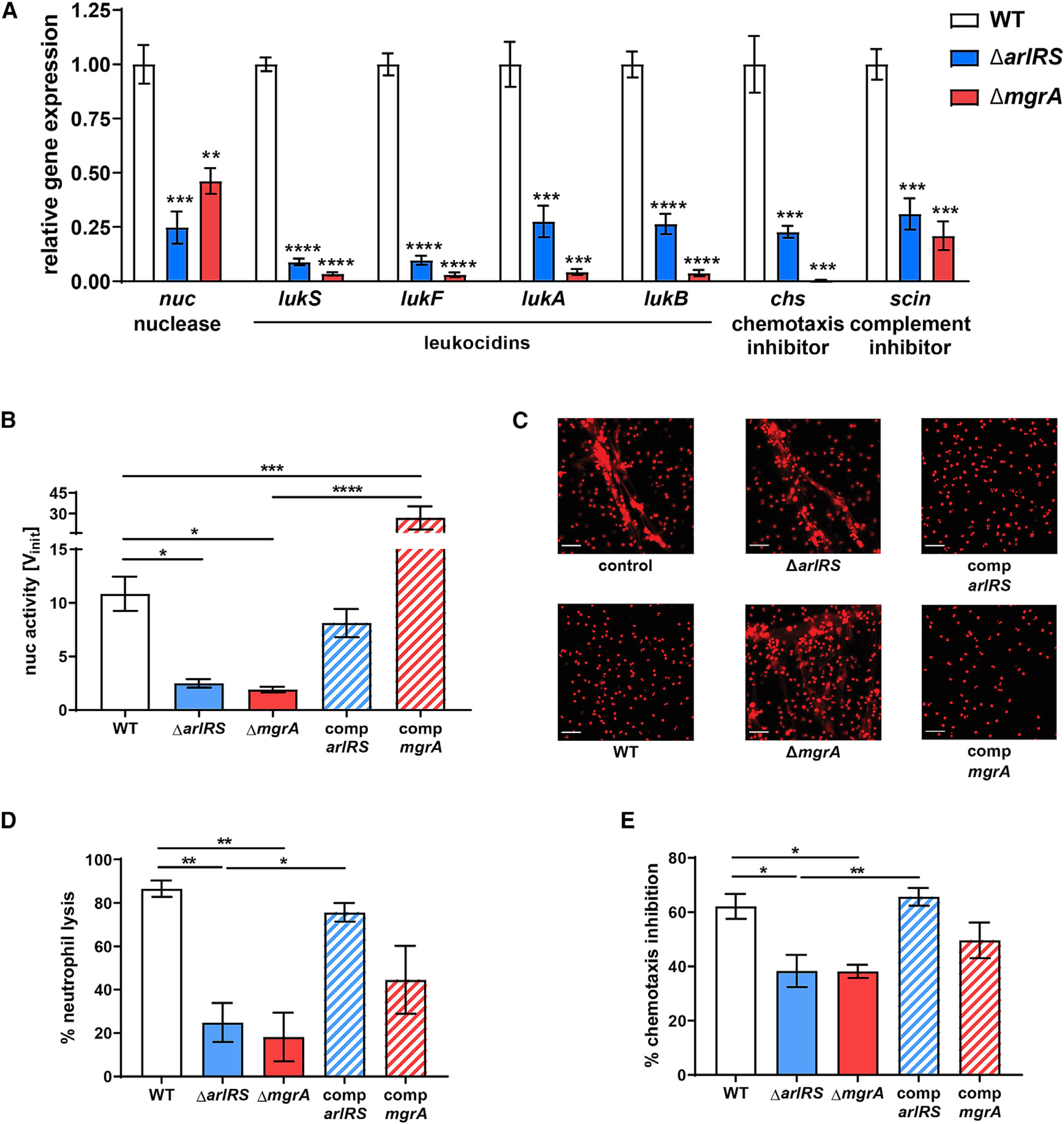
ArlRS and MgrA control innate immune evasion of *S. aureus* (A) Expression of immune evasion genes in mid-exponential *S. aureus* RPMI culture was measured with qPCR and normalized to *gyrB* expression. (B and C) Nuclease activity in culture supernatants (B) and their ability to digest NETs, visualized with PI (C), were measured. (D and E) The ability of *S. aureus* culture supernatants to kill human neutrophils (D) and block neutrophil chemotaxis (E) was measured. Representative images are shown. Scale bar, 100 μm. Data are shown as mean ± SEM. n = 3 (A), 3–6 (B), 4 (D), and 6 (E). *p < 0.05; **p < 0.01; ***p < 0.001; ****p < 0.0001. In (A), all p values for comparisons to WT. All significant p values between the groups are marked on graphs. See also Figure S5.

To understand mechanisms behind observed changes in immune evasion, we examined our earlier RNA sequencing (RNA-seq) data and noticed decreased expression of several immune evasion genes in the mutant strains (Crosby et al., 2020). We confirmed these RNA-seq data by directly measuring gene expression with qPCR. Expression of the nuclease, bicomponent leukocidins LukSF (Panton-Valentine leukocidin [PVL]) and LukAB, chemotaxis-inhibitory protein of *S. aureus* (CHIPS), and staphylococcal complement inhibitor (SCIN) were all dramatically reduced in the Δ*arlRS* and Δ*mgrA* mutants (Figure 3A).

### ArlRS and MgrA mutants cannot evade neutrophil NETs

Formation of NETs to ensnare the pathogens is an important host defense mechanisms in skin infections (Stephan and Fabri, 2015). Degradation of these NETs by *S. aureus* nuclease allows the pathogen to evade entrapment and subsequently transform degraded NET fragments into the macrophage-killing deoxyadenosine. Thus, nuclease is responsible for *S. aureus* immune evasion in abscesses and the spread of bacteria from the infectious foci in skin (Berends et al., 2010; Thammavongsa et al., 2013; Tseng et al., 2012; Wang et al., 2020; Yipp et al., 2012). We therefore investigated if the decreased *nuc* expression in the Δ*arlRS* and Δ*mgrA* mutants leads to their reduced ability to degrade NETs.

The nuclease activity in supernatants of the Δ*arlRS* and Δ*mgrA* mutants was significantly reduced (Figure 3B). This reduced activity was restored to the MRSA WT level after chromosomal complementation of *arlRS* and significantly exceeded the WT level when *mgrA* was complemented by expressing it with its native promoter from a complementing plasmid (Figure 3B). The reduced nuclease activity translated to a pronouncedly decreased ability of *S. aureus* to digest NETs. When stimulated human neutrophils created NETs, visible as cords and meshwork of extracellular DNA, the supernatant from MRSA WT readily destroyed these NETs, leaving behind only the nuclear DNA (Figure 3C). This activity was dependent on secretion of staphylococcal nuclease (Figure S5A). The supernatants from the Δ*arlRS* and Δ*mgrA* mutants, unlike the WT, did not destroy the NETs but regained the NET-degrading activity upon complementation of the missing genes (Figure 3C). The same pattern was confirmed by quantification of visible NETs with image-processing software (Figure S5B). Altogether, the results point to markedly decreased ability of the mutants lacking ArlRS and MgrA to evade the NETs.

### ArlRS and MgrA mutants fail to kill incoming neutrophils or prevent chemotaxis

Bicomponent leukocidins, a group of toxins targeting leukocytes, are produced by *S. aureu*s to lyse the incoming host immune cells (Lewis and Surewaard, 2018; Seilie and Bubeck Wardenburg, 2017; Spaan et al., 2017). This lytic activity can be detected in *S. aureus* culture supernatants and is dependent on synergistic activity of both LukAB and LukSF (PVL) (Figure S5C). Indeed, we observed that filtered spent media from MRSA WT was able to kill human neutrophils, and in contrast, spent media from the Δ*arlRS* and Δ*mgrA* mutants had significantly attenuated neutrophil killing (Figure 3D). This mutant phenotype correlated with their reduced expression of *lukSF* and *lukAB* genes seen in the qPCR analysis (Figure 3A). The ability of the Δ*arlRS* and Δ*mgrA* mutants to kill neutrophils was fully or partly restored by chromosomal complementation (Figure 3D).

As part of the immune evasion, *S. aureus* prevents neutrophil recruitment to the infection site (de Haas et al., 2004; de Jong et al., 2019; Lewis and Surewaard, 2018). Indeed, when we tested human neutrophil chemotaxis toward *N*-formyl-met-leu-phe (fMLP) chemoattractant peptide, we observed a chemotaxis-inhibiting activity of *S. aureus* spent media that was dependent on CHIPS (Figure S5D). The addition of supernatant from the MRSA WT strain (at a sub-lytic concentration causing no direct killing of assayed neutrophils) led to a marked inhibition of chemotaxis (Figure 3E). When ArlRS-MgrA cascade mutants were tested, they could not inhibit chemotaxis to the same extent as MRSA WT (Figure 3E), in accordance with their reduced expression of *chs* gene encoding the CHIPS chemotaxis-inhibitory protein (Figure 3A). Mutant strains had their chemotaxis-inhibitory ability restored upon chromosomal complementation of the missing *arlRS* and *mgrA* genes (Figure 3E).

### ArlRS and MgrA mutants are more susceptible to killing by neutrophil α-defensins and oxygen radicals

**Figure 4.**
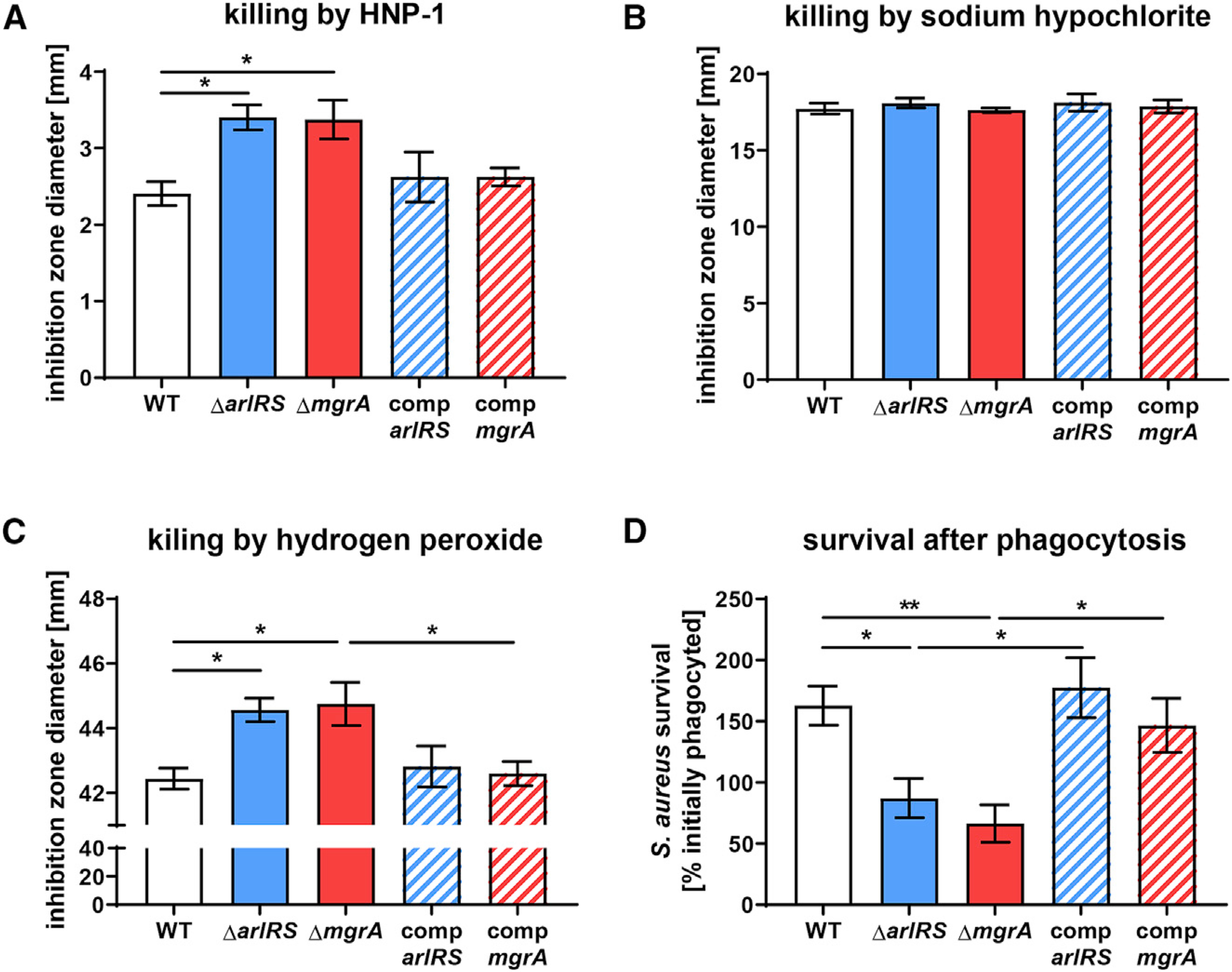
ArlRS and MgrA control *S. aureus* survival after neutrophil phagocytosis (A–C) Resistance of *S. aureus* to various compounds used by neutrophils to kill the bacteria, including human α-defensin HNP-1 (A), sodium hypochlorite (B), and hydrogen peroxide (C), was measured with agar diffusion assays. Additionally, survival of *S. aureus* 1 h after phagocytosis by human neutrophils was measured (D). Data are shown as mean ± SEM. n = 8 (A, C, and D) or 4 (B). *p < 0.05; **p < 0.01. All significant p values between the groups are marked on graphs.

Neutrophils directly kill *S. aureus* by producing antimicrobial peptides (like α-defensins) and ROS (mainly hypochlorite) (Brandt et al., 2018; Kobayashi et al., 2015; Lewis and Surewaard, 2018). When we measured resistance of MRSA to killing by one of human neutrophil α-defensins (HNP-1), the Δ*arlRS* and Δ*mgrA* mutants were significantly more susceptible than the WT strain, and this increased susceptibility was reversed by chromosomal complementation of the missing genes (Figure 4A). It has been previously theorized that Δ*mgrA* mutants might be more resistant to oxidative killing (Chen et al., 2006). Contrary to this hypothesis, we did not observe any changes in resistance to hypochlorite in the Δ*arlRS* and Δ*mgrA* mutants (Figure 4B). They were slightly but significantly more susceptible to killing by hydrogen peroxide, which could be reversed by chromosomal complementation of the missing genes (Figure 4C). In accordance with these findings, when survival inside human neutrophils was measured after phagocytosis, MRSA WT was not killed and even managed to proliferate, while the Δ*arlRS* and Δ*mgrA* mutants were killed (Figure 4D). Chromosomal complementation of the missing genes restored the ability of mutant strains to proliferate inside neutrophils (Figure 4D). This further explains the observed accelerated immune clearance of the infecting Δ*arlRS* and Δ*mgrA* mutants in infected skin.

### ArlRS and MgrA are needed for evasion of neutrophils in skin *in vivo*

**Figure 5.**
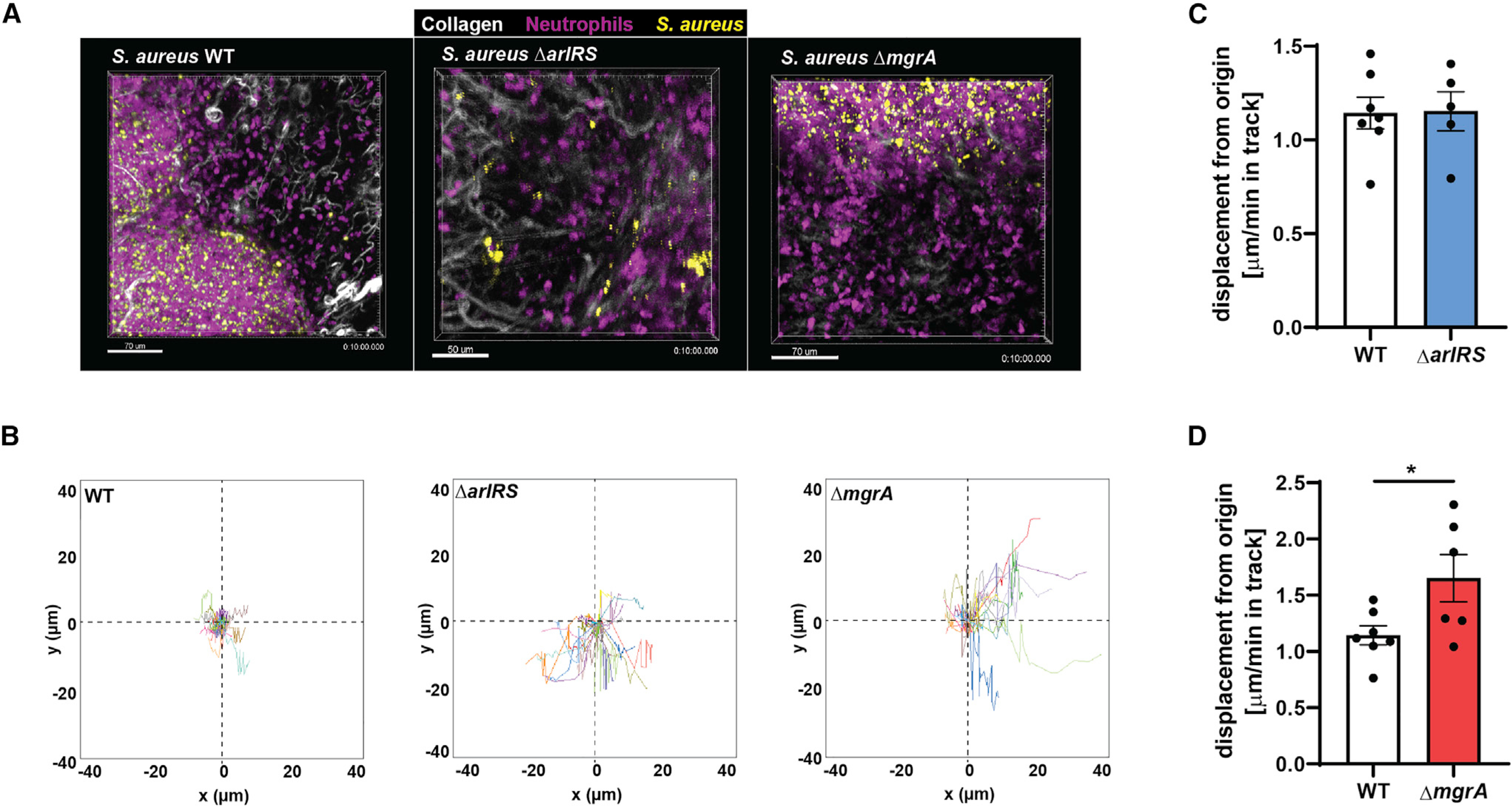
ArlRS and MgrA allow *S. aureus* to affect neutrophil movement during skin infection *in vivo* Multiphoton intravital microscopy was used to image neutrophil/*S. aureus* interactions *in vivo* for 10 min at 24 h post-infection. (A) Representative image taken from time-lapse videos showing neutrophils at the infection site from WT, Δ*arlRS*, and Δ*mgrA* skin infections. (B) Quantification of neutrophil track displacement length in the x-y position in *S. aureus* skin infections. (C and D) Quantification of the mean displacement of neutrophils per minute (velocity). Data are shown as mean ± SEM. n = 5–7. *p < 0.05. See also Videos S1 and S2.

**Figure 6.**
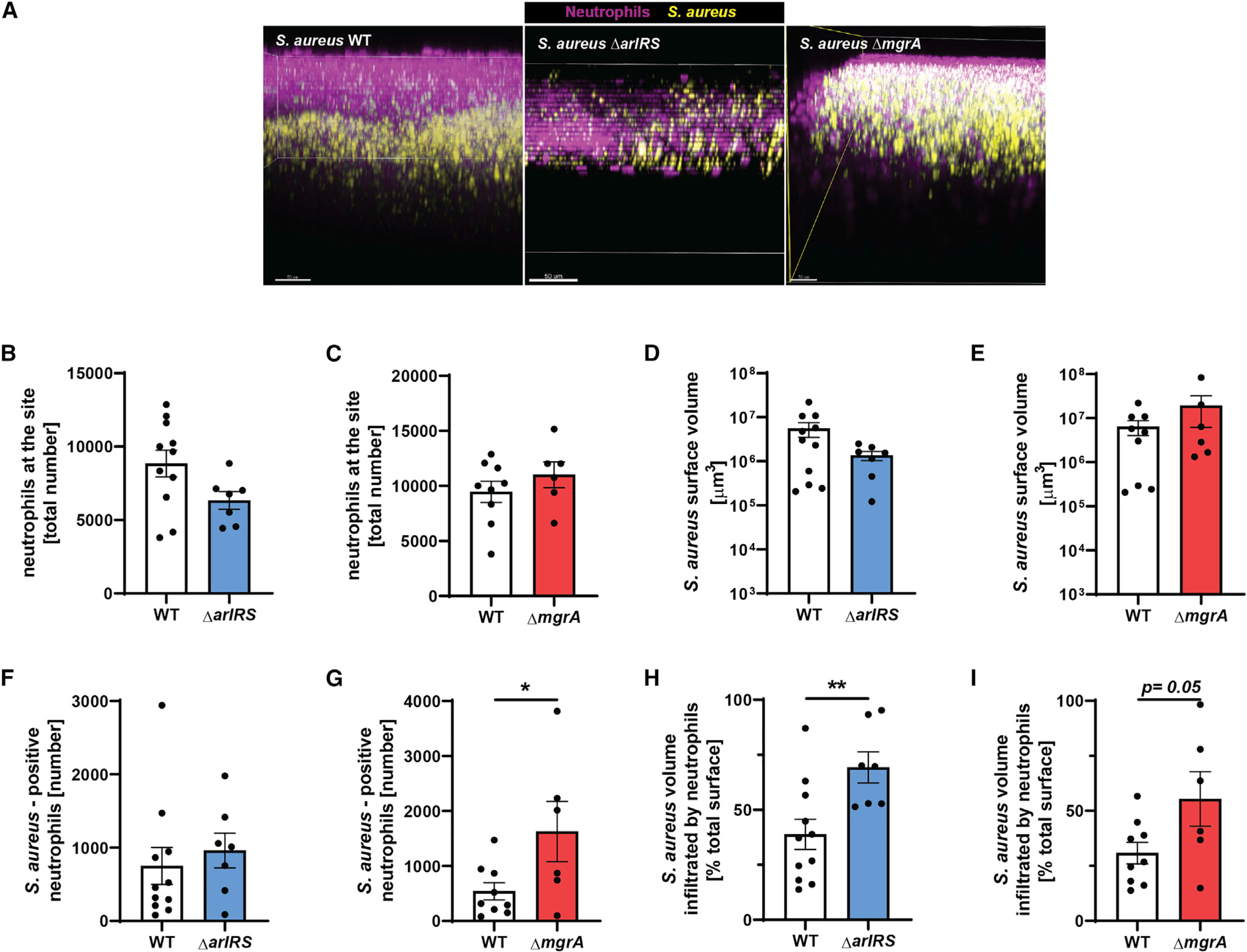
ArlRS and MgrA are needed for immune evasion during skin infection *in vivo* Multiphoton intravital microscopy was used to image neutrophil/*S. aureus* interactions *in vivo* at 24 h post-infection. (A) Representative intravital image showing a three-dimensional stitched image viewed from the x-z plane (side view) showing neutrophil localization at the infection site. (B and C) Image analysis quantification of total neutrophil spots at the infection site. (D and E) Total *S. aureus* surface volume at the infection site. (F and G) *S.*-*aureus*-positive neutrophils. (H and I) Percentage of *S. aureus* volume that was infiltrated by neutrophils. Data are shown as mean ± SEM. n = 6–11. *p < 0.05; **p < 0.01. See also Videos S3 and S4.

To observe the ongoing immune evasion during skin infection, we used multiphoton intravital microscopy to visualize interactions between *S. aureus* and neutrophils *in vivo* (Figure 5). Neutrophil behavior was tracked using the Catchup^IVM-red^ reporter mouse after infection with MRSA WT or the Δ*arlRS* and Δ*mgrA* mutant strains at 24 h post-infection (Figure 5A). We observed the most profound phenotypic differences when the Δ*mgrA* mutant was used. Neutrophils from mice infected with the Δ*mgrA* mutant displayed longer track lengths (Figure 5B; Videos S1 and S2) and an overall increase in displacement over time (velocity) compared to neutrophils from mice infected with MRSA WT (Figure 5D). We then analyzed neutrophil 3D localization at the infection site by applying a surface reconstruction on the neutrophil and *S. aureus* channels (Figure 6A; Videos S3 and S4). Both MRSA WT and the mutants recruited the same number of neutrophils to the general infection site (Figures 6B and 6C), and the total surface volume of *S. aureus* at the visualized site did not differ between the WT and mutant strains (Figures 6D and 6E). There were, however, marked differences in the way neutrophils behaved toward bacteria within the infection site. Significantly more neutrophils directly interacted with *S. aureus* in the mice infected with Δ*mgrA* mutant (Figure 6G), resembling the earlier *in vitro* observations (Figure 2E). Furthermore, there was a strong trend toward neutrophils infiltrating into the *S. aureus* layer in Δ*mgrA*-infected mice (Figure 6I). The Δ*arlRS* mutant generally presented an intermediate phenotype between the WT and the Δ*mgrA*, but a significantly increased infiltration of mouse neutrophils into the *S. aureus* layer also occurred in the Δ*arlRS* strain (Figure 6H), showing that also this element of the regulatory cascade is needed for efficient evasion of neutrophil attacks. Overall, while MRSA WT was able to limit neutrophil movement and prevent them from directly engaging the growing bacterial community in skin, the strain lacking MgrA (and, to a smaller degree, the strain lacking ArlRS) was unable to induce such immune evasion. These observations from live imaging of *in vivo* infection confirm the findings from *in vitro* systems.

## DISCUSSION

Skin is the most common site of *S. aureus* infection and the most common foci of systemic spread. The interplay of bacteria with the immune system dictates the extent of these two events. In its fight against the host’s immune system, *S. aureus* relies on timely and precisely regulated production of its virulence factors. In this study, we aimed to identify the regulatory system responsible for controlling these diverse direct and indirect mechanisms of immune evasion.

**Figure 7.**
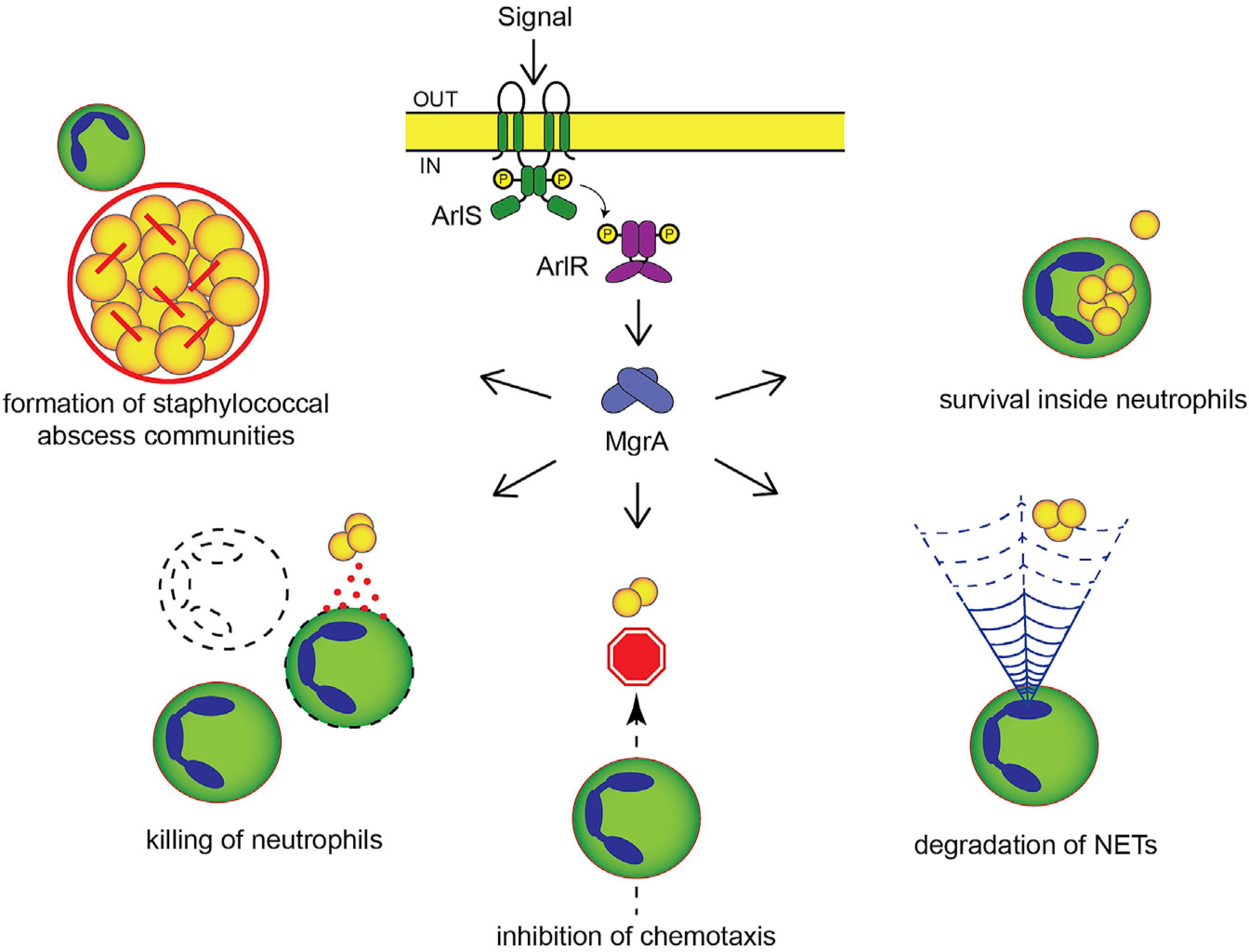
Proposed model of innate immune evasion control by ArlRS and MgrA during *S. aureus* infection With a functional ArlRS-MgrA cascade, the initial signal detected by the ArlRS two-component system induces expression of the global regulator MgrA, which in turn controls expression of various genes involved in virulence and immune evasion. By suppressing expression of large surface proteins with anti-adhesive properties (Ebh and SraP), the active cascade allows *S. aureus* to bind fibrinogen and form tight three-dimensional abscess communities where bacteria are shielded from phagocytes. Active cascade also causes *S. aureus* to secrete various immune evasion factors, such a leukocidins (LukAB and LukSF), CHIPS, SCIN, and nuclease, which together act to kill incoming neutrophils, prevent their chemotaxis and movement, and digest NETs used by neutrophils to ensnare bacteria. Finally, due to the cascade’s involvement with *S. aureus* resistance to antimicrobial peptides and, to a smaller degree, oxygen radicals, active ArlRS and MgrA promote bacterial survival inside neutrophils after phagocytosis. See also Figure S6.

Using a combination of mouse infection models, intravital microscopy, and *in vitro* models of isolated pathogenic processes, we discovered that the ArlRS-MgrA regulatory cascade controls *S. aureus* virulence in skin and that this is largely due to its regulation of the staphylococcal immune evasion (outlined in Figure 7). ArlRS and MgrA directly controlled expression of immune evasion factors (nuclease, LukSF, LukAB, CHIPS, and SCIN) and affected resistance to killing by neutrophil antimicrobial peptides and ROS. It also controlled the spatial organization of the *S. aureus* abscesses, which affected the pathogens’ ability to hide from immune attacks. Thus, our results identify the ArlRS-MgrA regulatory cascade as being central to *S. aureus* skin virulence and immune evasion. This adds ArlRS and MgrA to the short list of skin virulence regulators (Agr, SaePQRS, and CodY) and expands other reports of ArlRS or MgrA being involved in systemic infections, such as staphylococcal sepsis, endocarditis, arthritis following bacteremia, and muscle infection (Benton et al., 2004; Chen et al., 2009; Crosby et al., 2016b; Gupta et al., 2013; Jonsson et al., 2008; Li et al., 2019; Liu et al., 2014; Radin et al., 2016; Walker et al., 2013). The significance of the ArlRS-MgrA cascade across so many models, both local and systemic, points to it being one of the most important *S. aureus* regulatory systems for survival in the host. Even though the signal activating ArlRS signaling is still unknown, our data indicate that ArlRS-MgrA is functionally active inside the host’s skin, allowing *S. aureus* to mount an appropriate immune evasion response.

Abscess formation is an active process, controlled both by the host and by the invading *S. aureus* (Cheng et al., 2011; Kobayashi et al., 2015). The characteristic structure of *S. aureus* abscesses includes the SAC in the center, encased in protective layers of fibrinogen and polymerized fibrin, and surrounded by further layers of tissue debris, dead and living neutrophils, and macrophages at the periphery (Brandt et al., 2018; Cheng et al., 2011; Kobayashi et al., 2015). *S. aureus* virulence factors are also not randomly distributed within abscess but accumulate at distinct parts of the abscess structure (Cheng et al., 2009; Guggenberger et al., 2012). Formation of the central SAC (and the abscess in general) is thought to protect bacteria from attacks of host phagocytes and to create a niche for staphylococcal persistence. *S. aureus* mutants lacking ArlRS or MgrA components of the regulatory cascade failed to form the usual SAC in the center of the skin abscesses, instead producing a disordered spread of individual cells throughout the entire abscess. As shown before in *in vitro* models, lack of a typical spatial structure of the abscess could leave individual bacteria exposed to neutrophil phagocytosis (Guggenberger et al., 2012). This probably contributed to the accelerated clearance and the failure of Δ*arlRS* and Δ*mgrA* mutant strains to persist. When we investigated the mechanism of this altered spatial organization in *in vitro* model, it became apparent that it was caused by inability of bacteria to bind host fibrinogen, which acted as an organizing agent for the model SAC, crosslinking individual bacteria to form a tight, fibrinogen-encased three-dimensional structure. This is consistent with previous reports that de-repression of giant surface proteins with interfering activity in the Δ*arlRS* and Δ*mgrA* mutants leads to failed clumping and fibrinogen attachment by *S. aureus* (Crosby et al., 2016b; Kwiecinski et al., 2014). Indeed, the de-repression of the largest of these giant surface proteins (Ebh) prevented formation of tightly clumped *S. aureus* communities in our three-dimensional model of SAC. The exposure of invading *S. aureus* Δ*arlRS* and Δ*mgrA* mutants to immune attacks was possibly further exacerbated by mutants’ failure to adhere to dermal fibroblasts, preventing bacteria from using the intracellular niche to evade phagocytosis. We demonstrated that this phenotype, previously noted for adhesion to endothelial cells (Kwiecinski et al., 2019; Li et al., 2019; Seidl et al., 2018), was caused by the de-repression of the giant surface proteins SraP and Ebh. The overall observed anti-virulence effects of the de-repressed giant surface proteins, which interfere with typical *S. aureus* microbial surface components recognizing adhesive matrix molecules (MSCRAMMs) binding to their ligands (Kwiecinski et al., 2019), is consistent with the known importance of the MSCRAMMs in skin infection (Kwiecinski et al., 2014). These spatial and organizational anomalies caused by the altered regulation of giant surface proteins expose infecting *S. aureus* to immune attacks (Figure S6). Our findings highlight the importance of abscess three-dimensional structure for the outcome of *S. aureus* skin infections and identify ArlRS and MgrA as the key regulators of the abscess structuring.

At the site of infection, neutrophils kill bacteria through production of ROS and secretion of toxic compounds like antimicrobial peptides. We demonstrated that Δ*arlRS* and Δ*mgrA* mutant strains are more susceptible to neutrophil α-defensins, in accordance with earlier observation of functional MgrA being necessary for upregulation of protective *mprF* and *dltA* in response to antimicrobial peptide challenge (Li et al., 2019; Matsuo et al., 2010). We also show that lack of ArlRS and MgrA causes small but statistically significant increase in susceptibility to killing by hydrogen peroxide. This might seem counterintuitive, because MgrA was identified as oxidation-sensing molecule in *S. aureus*, and it was speculated that its absence in a mutant strain would lock the cell into a permanent oxidation-responsive state (Chen et al., 2006). However, other reports indicated that genes directly involved in survival of oxidative stress are regulated not by MgrA but rather by its homolog, SarZ (Chen et al., 2009). Functional MgrA was even shown to be necessary for a correct response to nitric oxide stress (Favazzo et al., 2019). A 2- to 3-fold decrease in expression of staphyloxanthin pigment synthesis genes (responsible for *S. aureus* resistance to ROS) was also reported in absence of ArlRS or MgrA (Crosby et al., 2020). Altogether, it appears that ArlRS-MgrA cascade is involved to some degree in protection of *S. aureus* from ROS, though the exact mechanism remains unknown.

In addition to direct killing, neutrophils can ensnare and kill the invading pathogens through the production of NETs, which prevent the spread of bacteria from skin (Stephan and Fabri, 2015; Tseng et al., 2012; Yipp et al., 2012). The nuclease of *S. aureus* allows it to evade these NETs by digesting their DNA backbone, liberating individual bacteria from the trap (Berends et al., 2010). Failure to produce nuclease in Δ*arlRS* and Δ*mgrA* mutants therefore likely contributed to their accelerated immune clearance from the infected skin in our model, adding yet another mechanism to immune evasion control by the ArlRS-MgrA cascade. Considering this increased evasion of NETs-mediated killing and direct killing by defensins and ROS, combined with the previously described involvement of the ArlRS in *S. aureus* resistance to the neutrophil-induced manganese starvation (Radin et al., 2016), it is evident that the ArlRS-MgrA cascade is necessary for evasion of nearly all types of neutrophil attacks.

Even more important than survival of direct killing by neutrophils is the *S. aureus* ability to avoid altogether attacks by host phagocytes. *S. aureus* achieves it by production of immune evasion molecules preventing neutrophils from approaching the infectious foci and killing phagocytes that nevertheless get too close. As we demonstrated, many of these evasion molecules, such as the neutrophil-killing bicomponent leukocidins (Lewis and Surewaard, 2018; Seilie and Bubeck Wardenburg, 2017; Spaan et al., 2017), as well as chemotaxis and complement inhibitors CHIPS and SCIN (de Haas et al., 2004; Rooijakkers et al., 2005), are all regulated by the ArlRS and MgrA system. We observed substantially decreased production of the two leukocidins (LukSF and LukAB) in the Δ*arlRS* and Δ*mgrA* mutants, leading to inability of these mutants to kill human neutrophils. This is consistent with previous suggestions that ArlRS might be involved in regulation of leukocidin expression (Crosby et al., 2020; Harper et al., 2018; Párraga Solórzano et al., 2019). Also, expression of both CHIPS and SCIN had decreased in the Δ*arlRS* and Δ*mgrA* mutant strains. All this further supports the notion of ArlRS and MgrA being central for immune evasion.

Notably, many of the mentioned immune evasion molecules are human specific. LukSF does not kill mouse neutrophils, while LukAB has only very weak killing ability (Spaan et al., 2017), though *S. aureus* mutants lacking these leukocidins previously show phenotypes in animal skin infection models, suggesting their limited activity might still play some role (Lacey et al., 2016; Seilie and Bubeck Wardenburg, 2017; Spaan et al., 2017). Similarly, SCIN is inactive, and CHIPS is less active in mouse than in human infections (de Haas et al., 2004; Rooijakkers et al., 2005). This indicates that the Δ*arlRS* and Δ*mgrA* mutants would present a much stronger phenotype in a real human infection than the one we observed in our mouse model. This is supported by our observation of a very profound phenotype in the *in vitro* abscess model with human neutrophils.

To further ascertain our findings, we visualized the real-time *S. aureus* interaction with neutrophils in the infected skin. Direct visualization of the infectious process inside a living organism, with all its complex multicellular interactions, provides unparalleled possibility to explore mechanistic details and confirm conclusions extrapolated from *in vitro* or whole-animal experiments (Scott et al., 2019). Observation of neutrophil behavior in skin infected by the WT strain and its counterparts lacking ArlRS and MgrA showed striking differences. On the large scale, both WT MRSA and the mutant strains caused similar recruitment of neutrophils to the infected area. The behavior of the neutrophils in the direct vicinity of the bacteria, however, where immune evasion strategies of *S. aureus* are acting, showed marked differences. While neutrophils in the WT skin infection had reduced motility and did not engage bacteria directly, in the Δ*mgrA* mutant infection, neutrophils presented a more actively motile phenotype and were able to access *S. aureus*. This failure of immune evasion was most evident in the mutant lacking MgrA, but *S. aureus* lacking ArlRS also could not prevent neutrophils from penetrating inside its colony. The decreased mobility and failure to enter into *S. aureus* community probably resulted from a combination of the protective fibrinogen layer on surface of bacterial community, decreased neutrophil chemotaxis, and neutrophil damage caused by staphylococcal toxins. As many of the immune evasion molecules of *S. aureus* are human specific or require high concentration to be active in mouse, it is possible that phenotypes in this model depended largely on the altered structure of bacterial community and fibrinogen deposition and that the anti-chemotactic and neutrophil-killing activities were limited to the area inside and immediately in contact with the *S. aureus* colony, where concentrations of the immune evasion molecules were the highest. In real-life settings of human infection, we would expect a much more striking combined effect of anti-chemotactic, anti-phagocytic, and neutrophil-killing mechanisms. Despite these reservations, the observation of an overall functional failure of immune evasion during the ongoing skin infection by the cascade mutants confirms our conclusions from the *in vitro* models.

The structure of the cascade (Figure 7), with MgrA being its final effector, expressed at a low basal level even in the absence of the ArlRS two-component system (Crosby et al., 2016b; 2020), indicates that phenotypes of the Δ*arlRS* and Δ*mgrA* mutants should be overlapping, but with a more pronounced phenotype in the Δ*mgrA* strain. This was indeed the case in majority of the experiments (including mouse infection), but in a few cases, the phenotype of the Δ*arlRS* strain was more pronounced. This might be due to activity of another global regulator, Spx, also controlled by the ArlRS. However, Spx is responsible for response to beta-lactams and stress and has not been linked to any of the virulent phenotypes in question (Bai et al., 2019; Crosby et al., 2020). It is more likely that the ArlRS-MgrA signaling cascade includes additional undescribed levels of signal integration. Notably, the bulk of our knowledge about the ArlRS-MgrA cascade comes from experiments conducted in rich laboratory bacteriological media under optimal conditions. Our observations indicate that under different environmental conditions, additional regulatory elements are possibly interacting with the cascade.

In conclusion, our work identified the regulatory cascade of ArlRS and MgrA as one of the main regulators involved in *S. aureus* skin infection, particularly in the development of abscess structure, the interaction with host cells, and evasion of the host immune response. The importance of the ArlRS and MgrA regulatory cascade for skin infections makes it a particularly promising drug target and an alternative to targeting individual virulence factors. By interfering with just this single cascade, one could block multiple and diverse immune evasion mechanisms, rendering *S. aureus* defenseless against host attacks. Further disentangling of different parts of *S. aureus* virulence regulation, identification of the relative contribution of individual virulence factors, and understanding of the overlap among host protein binding, abscess structure, and immune evasion will hopefully lead to not only a better understanding of *S. aureus* biology but also novel treatment strategies.

## STAR★METHODS

### RESOURCE AVAILABILITY

#### Lead contact

Further information and requests for resources and reagents should be directed to and will be fulfilled by the lead contact, Alexander R. Horswill (alexander.horswill@cuanschutz.edu).

#### Materials availability

All unique/stable reagents generated in this study are available from the lead contact and, in some instances, may require a completed materials transfer agreement.

#### Data and code availability

- This paper does not report original datasets.
- This paper does not report original code.
- Any additional information required to reanalyze the data reported in this paper is available from the lead contact upon request.

### EXPERIMENTAL MODEL AND SUBJECT DETAILS

#### Bacterial strains and plasmids

Bacterial strains and plasmids are listed in the key resources table. *S. aureus* was grown in tryptic soy broth (TSB) at 37°C with shaking, or in Roswell Park Memorial Institute medium 1640 (RPMI) at 37°C and 5% CO_2_ with shaking. For CFU counts, samples were serially diluted, plated on tryptic soy agar (TSA), and colonies counted after incubation at 37°C. *L. lactis* was grown at 30°C, without shaking, in M17 broth with 0.5% glucose. When needed, antibiotics were added to media: chloramphenicol (Cm, 10 μg/ml), erythromycin (Erm, 10 μg/ml, or 5 μg/ml for *L. lactis*), tetracycline (Tet, 1 μg/ml), spectinomycin (Spc, 1000 μg/ml).

#### Mice

Male and female C57BL/6J mice were purchased from the Jackson Laboratories and were housed in groups of two to five in SPF ABSL-2 animal facility of University of Colorado Anschutz Medical Campus. Male and female Catchup^IVM-red^ mice (Hasenberg et al., 2015) were bred in the CCMG facility at the University of Calgary Cumming School of Medicine, and were housed in groups of two to five. All mice were provided with nesting material for enrichment. Mice were 8–10 week (C57BL/6J mice) or 7–8 week (Catchup^IVM-red^) old when they were used for experiments, and were randomly assigned to experimental groups. At the experiments’ endpoints, mice were euthanized according to local guidelines. Animal experiments were approved by the University of Colorado Institutional Animal Care and Use Committee (protocol 00486) and by the University of Calgary Animal Care Committee (protocol AC19–0138).

#### Human blood collection

Human heparin-anticoagulated whole blood was collected from anonymous adult volunteers of both sexes and used directly for experiments, or centrifuged at 2000 *g* for 20 min to obtain plasma. Blood collection was approved by the Colorado Multiple Institutional Review Board (protocol 17–1926).

#### Mouse dermal fibroblasts

Dermal fibroblasts were isolated from combined ear skin of three 8-week old female C57BL/6J mice and were cultivated in RPMI medium with fetal calf serum, asparagine, glutamine, 2-mercaptoethanol, and penicillin-streptomycin supplementation as described previously (Khan and Gasser, 2016).

### METHOD DETAILS

#### Construction of bacteria mutants

The Δ*agr*::tetM, Δ*arlRS*::tetM, *chs*:: ϕNΣ, *lukA*:: ϕNΣ, and *sraP*::ϕNΣ (ϕNΣ transposons from Nebraska Transposon Library(Fey et al., 2013)) mutation cassettes were transduced between *S. aureus* strains with phage 80α or 11, as described previously (Novick, 1991). The *lukS*:: ϕNΣ^spc^ mutant was created by exchanging Erm resistance cassette in the corresponding ϕNΣ Nebraska Transposon Library mutant for Spc resistance, and transducing it into the WT strain (Bose et al., 2013). The Δ*sak*::tetM mutant was created using pJB38 deletion plasmid as described before (Kwiecinski et al., 2019), with regions flanking the *sak* amplified using primer pairs JK41/JK42 and JK43/JK44 (Table S1), and the constructed cassette was afterward transduced with phage 11.

#### Mouse skin infection

Previously described murine skin infection model was used (Grundstad et al., 2019; Kwiecinski et al., 2014). *S. aureus* from mid-log growth phase in TSB was washed with phosphate-buffered saline (PBS), and resuspended for infection in a sterile saline. Abdomens of mice were shaved with a microtome blade, wiped with alcohol pads, and 50 μl of bacteria suspension, containing either 1 × 10^8^ CFU (for necrotic lesion scoring) or 1–2 × 10^6^ CFU (for histology and skin CFU count), were injected subcutaneously through an insulin syringe. Developing lesions were photographed daily, and lesion area was measured with the FIJI software. At pre-determined days mice were euthanized, and the infected skin area was either excised, fixed with a phosphate-buffered 4% formaldehyde (4% PBF), embedded in paraffin, sliced, and stained with hematoxylin and eosin or with a modified tissue gram stain (Becerra et al., 2016), or an 8 mm diameter punch biopsy of the infected area was taken, homogenized, and used for skin CFU counts. The skin histopathology slides were assessed for the abscess area (measured with the FIJI) and the presence of staphylococcal abscess communities by an investigator unaware of the experimental groups.

#### *In vitro* staphylococcal abscess community model

A model of *S. aureus* growing in a 3-dimensional collagen/fibrin(ogen) gel (Guggenberger et al., 2012) was used to model behavior of the staphylococcal abscess community in the skin matrix. Mid-log phase bacteria were suspended in 1.7 mg/ml rat type I collagen solution in RPMI, pH 7.4, at 1 × 10^5^ CFU/ml, and 10 μl of the solution was allowed to solidify for 45 min in wells of an “angiogenesis μ-slide” chamber at 37°C, 5% CO_2_. Afterward, gel was overlaid with 50 μl of RPMI containing 3 mg/ml human fibrinogen and 5% human plasma, incubated for 16 h at 37°C, 5% CO_2_, and communities growing inside the gel matrix were afterward imaged with an Eclipse TE2000-E microscope or BZ-X710 microscope. Same procedure was followed for *L. lactis*, except that 30°C was used for incubation, and gels were stained with 5 μM Syto9 dye to help bacteria visualization.

#### Neutrophil isolation

Peripheral blood polymorphonuclear leukocytes (PMNs) were isolated from blood of healthy adult human volunteers using Polymorphprep density gradient (Oh et al., 2008), resulting in approximately 95% pure preparation (assessed with Wright-Giemsa stain), and were suspended in RPMI with 2% human serum albumin (RPMI/HSA) for subsequent assays.

#### Neutrophil challenge of *in vitro* abscess model

After 16 h growth of *S. aureus* gel abscess models, the medium above the gels was aspirated, wells washed with PBS, and filled with 50 μl of neutrophils in RPMI/HSA at 3 × 10^5^ PMNs/well. Before addition, neutrophils were stained with carboxyfluorescein diacetate succinimidyl ester (CFDA-SE). After 3 h incubation at 37°C, 5% CO_2_, propidium iodide was added in order to stain lysed cells and extracellular DNA, and the wells were imaged with BZ-X710 microscope.

#### Blood survival

To measure *S. aureus* survival in whole human blood, 50 μl of mid log-phase bacteria suspension in PBS containing 2.5 × 10^6^ CFU was added to 450 μl of human whole blood, and incubated on rotating platform at 37°C for 1 h. Afterward, blood was mixed with 500 μl of PBS with 0.5% saponin, 200 U/ml streptokinase, 100 μg/ml trypsin, 2 U/ml DNase I to lyse cells and break bacterial clumps, surviving CFU counted, and expressed as % of original inoculum (Thomer et al., 2016). As survival of MRSA WT varied from 10% to 70%, depending on a blood donor, surviving % of mutant strains was normalized to WT survival in each donor.

#### Co-culture with neutrophils

Neutrophils in 100 μl RPMI/HSA were added at 3 × 10^5^ cells /well to 96 well cell culture plates precoated for 1 h at 37°C with 50% human serum in PBS, and were allowed to settle for 15 min at a room temperature. Afterward, 100 μl of RPMI/HSA with 3 × 10^5^ CFU of mid-log phase *S. aureus* was added to wells (MOI = 1), and to synchronize neutrophil response, the plate was centrifuged for 7 min at 500 *g*, 4°C. The plate was subsequently incubated at 37°C, 5% CO_2_, and at the predetermined time points 22 μl of 1% saponin were added per well to lyse all neutrophils, and the viable *S. aureus* CFU in the well was counted (Lu et al., 2014).

#### qPCR experiments

For quantitative PCR (qPCR) analysis, MRSA strains were grown in RPMI to a mid-log phase, and their RNA was isolated and transcribed to cDNA as described before (Kwiecinski et al., 2019). qPCR was performed by amplifying 20 ng of cDNA in 20 μl total reaction volume with iTaq Universal SYBR Green Supermix in CFX96 Touch Real-Time PCR System, under the following conditions: 3 min at 95°C, 40 cycles of 15 s at 95°C and 30 s at 55°C, followed by a dissociation curve. “No template” and “no reverse transcription” controls were performed in parallel. Primers for qPCR are listed in Table S1, and primer pairs efficiencies were 85% (*ebh*), 93% (*sraP*), 100% (*nuc*), 86% (*lukS*), 92% (*lukF*), 91% (*lukA*), 93% (*lukB*), 93% (*chs*), 91% (*scin*), and 88% (*gyrB*). Data were analyzed and Cq determined with CFX manager. Expression was normalized to that of *gyrB*, and values represent three biological replicates.

#### Nuclease activity

Supernatants from 16–18h *S. aureus* cultures in RPMI were used to quantify nuclease activity using the previously described Förster resonance energy transfer (FRET) assay (Kavanaugh et al., 2019). To be in the linear range of the assay, supernatants were diluted 100 × with distilled water. Nuclease activity was expressed as the initial rate of the DNA cleavage reaction (V_init_).

#### NETs degradation

To visualize degradation of neutrophil extracellular traps (NETs) by supernatants from 16–18h *S. aureus* cultures in RPMI, a previously described method was used (Schilcher et al., 2014). Neutrophils in RPMI/HSA were seeded into “μ-slide 8 well” coverslip chambers at 2 × 10^5^ cells per 1 cm^2^, and stimulated with 25 nM phorbol 12-myristate 13-acetate (PMA) for 90 min at 37°C, 5% CO_2_, to induce NET formation. Afterward, culture supernatants diluted 20 × in RPMI were added to the chambers and incubated for 30 min at 37°C, 5% CO_2_, to allow degradation of NETs. Chambers were fixed with 4% PBF for 15 min, DNA was stained with 20 μM propidium iodide, and slides were imaged with the BZ-X710 microscope. To quantify amount of remaining NETs, in another set of experiments fixation with PBF was omitted in order for propidium iodide to stain exclusively the extracellular DNA in NETs and the cells undergoing lysis or NET secretion, and stained % of total area of random fields of view was measured with the FIJI software.

#### Killing of neutrophils

To measure killing of neutrophils by supernatants from 16–18h *S. aureus* cultures in RPMI, a previously described assay was used (Dumont et al., 2011). Neutrophils were seeded at 1 × 10^5^ cells per well into 96-well plate in 90 μl of RPMI/HSA, and 10 μl of bacterial supernatants were added (final concentration of 10%). After 3h incubation at 37°C, 5% CO_2_, the plates were centrifuged at 250*g*, 10 min, and resulting supernatants were used to measure lactate dehydrogenase (LDH) leakage from damaged cells as the marker of neutrophil lysis with an LDH Cytotoxicity Detection Kit. The % neutrophil lysis was calculated using neutrophils incubated with 10% of RPMI as “0% lysis” control, and incubated with 0.2% Triton X-100 as “100% lysis” control.

#### Chemotaxis inhibition

To measure inhibition of neutrophil chemotaxis by supernatants from 16–18 h *S. aureus* cultures in RPMI, an under-agarose chemotaxis method was used (Hii et al., 2004). Two 2 mm diameter wells were punched 5 mm apart in 0.5% agarose/RPMI gel, and filled with 5 μl of 1 × 10^7^ neutrophils in RPMI/HSA with 5% of bacterial supernatants (first well) or with 5 μl of 5 × 10^−7^ M N-Formyl-Met-Leu-Phe (fMLP) chemoattractant peptide (second well). After 90 min at 37°C, 5% CO_2_, images were taken with the BZ-X710 microscope, and the distance traveled by neutrophils under the agarose from the border of their well toward the well with the fMLP was measured. The % inhibition of chemotaxis was calculated in comparison to neutrophils mixed with 5% RPMI instead of supernatant (positive chemotaxis control). The supernatant concentration used (5%, 90 min incubation) was sub-lytic, confirmed by aspiration of neutrophils remaining in the well after the experiment, and staining them with trypan blue dye for viability measurement, consistently showing above 90% viability.

#### S. aureus susceptibility to α-defensins and oxygen radicals

Agar radial diffusion assays were used to measure susceptibility of the *S. aureus* from mid-log TSB culture. Agar overlay technique was used to detect zones of *S. aureus* growth inhibition caused by human α-defensin neutrophil peptide 1 (HNP-1) (Lehrer et al., 1991). Standard EUCAST disk diffusion susceptibility testing method (version 8.0) was used to detect susceptibility to ROS, except that instead of using antimicrobial disks, a 5 mm dimeter whole was punched in agar plates and filled with 10 μl of 10% hydrogen peroxide, or 20 μl of sodium hypochlorite solution at concentration equal to 2.5% active chlorine.

#### S. aureus survival after phagocytosis by neutrophils

A previously described method to measure intraphagosomal killing of *S. aureus* by neutrophils in suspension was adapted for adherent neutrophils (Pang et al., 2010). Human neutrophils in Hank’s Balanced Salt Solution with calcium and magnesium, 10% human serum, and 1% human serum albumin (HBSS^+++^) were placed in 48-well cell culture plate at 250 μl containing 1 × 10^5^ cells per well, centrifuged at 300 *g* for 3min, and allowed to adhere to well bottom for 30 min at 37°C. *S. aureus* from mid-log phase was opsonized by incubation in HBSS^+++^ for 15 min at 37°C, and was added to wells with neutrophils at MOI = 10 in 250 μl volume. The plates were centrifuged at 500 *g* for 5 min to put bacteria in a direct contact with the neutrophils, and were incubated at 37°C for 15 min to allow for phagocytosis. Afterward, wells were extensively washed to remove non-phagocyted *S. aureus*, filled with 250 μl fresh HBSS^+++^, and incubated at 37°C to allow for the killing of the ingested bacteria. After 1 h, the medium was aspirated from the wells, the neutrophils were lysed by filling the wells with 250 μl of 1% saponin, and viable CFUs were counted. The same lysis and counting procedure was performed on parallel wells immediately before the 1 h incubation to determine the baseline 100% viable CFU.

#### S. aureus adhesion to fibroblasts

Mouse dermal fibroblasts were grown to confluency in “μ-slide 8 well” coverslip chambers and washed with PBS. Mid-exponential phase *S. aureus* strains carrying sGFP-expressing plasmid were washed with PBS, resuspended in unsupplemented RPMI, and added to fibroblasts at MOI = 20. After 1h incubation at 37°C, 5% CO_2_, medium was aspirated, wells washed with PBS, adhered fluorescent bacteria in 5 random sites per each chamber were visualized with a BZ-X710 microscope, and % of area with adhered bacteria was measured with the FIJI software.

#### Intravital microscopy

Resonant-scanning multiphoton microscopy was used to image the skin of Catchup^IVM-red^ mice, in which neutrophils are tagged with a tdTomato red fluorescent protein (Hasenberg et al., 2015), and which was infected by subcutaneous injection of 5 × 10^6^ CFU of *S. aureus* strains expressing sGFP into a back flank. Mice were anaesthetized with xylazine and ketamine and a jugular catheter was inserted to maintain anesthesia as previously described (Yipp et al., 2012). Superfusion buffer (HBSS with no calcium, magnesium, nor phenol red) was then perfused across the exteriorized skin tissue to keep the skin moist at a flow rate set to 0.05.

The infected dermal skin tissue was imaged with a Leica SP8 2-photon microscope, equipped with 25 × 0.95 NA water objective lens, two InSight DeepSee pulsed infrared lasers (fixed 1040 nm and tunable 680–1300 nm) and a high speed 8 kHz resonant scanner. Laser excitation at 940 nm was used to excite tdTomato and GFP with external detectors (HyD-RLD2 BP 585/40 for tdTomato, HyD-RLD3 BP 525/50 for GFP) and second harmonic generation (external detector HyD-RLD4 BP 450/70) to visualize skin collagen. Laser power, detector settings and acquisition settings were maintained throughout each experiment.

#### Intravital image analysis

A 3D tile scan (4 × 4 fields of view; each field 350 × 350 × 100 μm^3^) was first collected to get an overview of the infection region. From there, 3 fields of view (350 × 350 × 50 μm^3^) were selected within the infection region to select the time-lapse positions. Images were collected every 30 s for 20 minutes to capture dynamic neutrophil behavior. The first 10 minutes of each video was analyzed using Imaris version 9.5.1 (Oxford Instruments). Pre-processing of videos included the MATLAB extension “normalize time points” to exclude voxels less than 1, and a manual stabilization to minimize translational drift. From there, neutrophil spots were detected with automated thresholding and tracks were detected with Brownian motion. The neutrophil X,Y,Z- position for each time point was exported and track displacement and velocity was analyzed in R.

Neutrophil spots from the 3D tile scan were detected using automated thresholding and default spot settings. *S. aureus* surface volume was detected using manual thresholding and specific threshold values was noted for each mouse. A masked channel was applied to the *S. aureus* surface and the “intensity max Ch-x(*S. aureus* mask)” filter was applied to quantify *S. aureus-*positive neutrophils (a measure of neutrophils interacting with *S. aureus*). Bacterial discovery was analyzed by creating a total neutrophil surface, applying a mask to the neutrophil channel and filtering the *S. aureus* surface as “intensity max Ch-y(neutrophil mask)” (a measure of infiltration of *S. aureus* layer by neutrophils).

### QUANTIFICATION AND STATISTICAL ANALYSIS

For each experiment, the number of replicates (N) and manner of data presentation (definition of center and precision of measures) is provided in figure legends. For all assays, data were pooled from at least two independent experiments. Differences between *S. aureus* strains were analyzed by ANOVA with a Dunnett’s multiple comparisons post-test (for comparison of mutant groups to WT strain) or Sidak’s multiple comparison post-test (for comparison of multiple groups with each other); by Kruskal-Wallis test with a Dunn’s multiple comparisons post-test (for CFU); by chi-square test with Bonferroni correction (for presence of SAC in abscesses); or by unpaired t test (for assays with only 2 groups). Two-tailed *p* values were calculated, and p < 0.05 was considered significant. Prism software was used for statistical calculations. For non-quantitative microscopy images, two independent experiments were performed and representative images are shown.

## Supplementary Material

1

2

3

4

5

## Figures and Tables

**KEY RESOURCES TABLE:** 

REAGENT or RESOURCE	SOURCE	IDENTIFIER

Bacterial and virus strains		

*S. aureus* USA300 CA-MRSA, erm^S^ (= LAC)	Boles et al., 2010	AH1263
*S. aureus* LAC Δ*agr*::tetM	Kiedrowski et al., 2011	AH1292
*S. aureus* LAC Δ*sak*::tetM	This Paper	AH4990
*S. aureus* LAC *nuc*::LtrB	Kiedrowski et al., 2011	AH1680
*S. aureus* LAC *chs*:: φNΣ	This Paper	AH4960
*S. aureus* LAC *lukA*:: φNΣ	This Paper	AH4963
*S. aureus* LAC *lukS*:: φNΣ^spc^	This Paper	AH4987
*S. aureus* LAC Δ*ebh*	Crosby et al., 2016b	AH3150
*S. aureus* LAC Δ*arlRS*	Walker et al., 2013	AH1975
*S. aureus* LAC Δ*arlRS* φ11::LL29tet *arlRS*	Kwiecinski et al., 2019	AH3244
*S. aureus* LAC Δ*arlRS* Δ*agr*::tetM	This Paper	AH3288
*S. aureus* LAC Δ*arlRS* Δ*sak*::tetM	This Paper	AH5599
*S. aureus* LAC Δ*arlRS*::tetM	Crosby et al., 2016b	AH3520
*S. aureus* LAC Δ*arlRS*::tetM Δ*ebh*	This Paper	AH3151
*S. aureus* LAC Δ*arlRS*::tetM *sraP*::φNΣ	This Paper	AH3817
*S. aureus* LAC Δ*arlRS*::tetM *sraP*::φNΣ Δ*ebh*	This Paper	AH3818
*S. aureus* LAC Δ*mgrA*::tetM	Crosby et al., 2016b	AH3455
*S. aureus* LAC Δ*mgrA*::tetM φ11::LL29erm *mgrA*	Crosby et al., 2016b	AH3485
*S. aureus* LAC Δ*mgrA*::tetM Δ*ebh*	Crosby et al., 2016b	AH3481
*S. aureus* LAC Δ*mgrA*::tetM *sraP*::φNΣ	Crosby et al., 2016b	AH3811
*S. aureus* LAC Δ*mgrA*::tetM *sraP*::φNΣ Δ*ebh*	Crosby et al., 2016b	AH3798
*S. aureus* LAC Δ*mgrA*	Crosby et al., 2016b	AH3375
*S. aureus* LAC Δ*mgrA* Δ*agr*::tetM	This Paper	AH4986
*S. aureus* ST5 MSSA (= 502A)	Parker et al., 2014	AH3610
*S. aureus* 502A Δ*arlRS*::tetM	Crosby et al., 2016b	AH3624
*S. aureus* 502A Δ*mgrA*::tetM	Crosby et al., 2016b	AH3625
*S. aureus* USA400 MRSA (= MW2)	Baba et al., 2002	AH843
*S. aureus* MW2 Δ*arlRS*::tetM	Crosby et al., 2016b	AH3060
*S. aureus* MW2 Δ*mgrA*::tetM	Crosby et al., 2016b	AH3456
*S. aureus* USA100 MRSA (= N315)	Kuroda et al., 2001	AH2398
*S. aureus* N315 Δ*arlRS*::tetM	Crosby et al., 2016b	AH3082
*S. aureus* N315 Δ*mgrA*::tetM	Crosby et al., 2016b	AH3473
*S. aureus* USA200 MSSA (= MN8)	Schlievert and Blomster, 1983	AH2413
*S. aureus* MN8 Δ*arlRS*::tetM	Crosby et al., 2016b	AH3063
*S. aureus* MN8 Δ*mgrA*::tetM	Crosby et al., 2016b	AH3480
*L. lactis*: surrogate host for ClfA expression	O’Brien et al., 2002	MG1363

Biological samples		

Whole blood, human	volunteers	N/A
Plasma, human	volunteers	N/A
Serum, human	Innovative Research	Cat# ISER10ML

Chemicals, peptides, and recombinant proteins		

Serum albumin, human	Millipore Sigma	Cat# 12667
Fibrinogen, human	Millipore Sigma	Cat# F3879
Polymorphprep	Accurate Chemical	Cat# AN1114683
Syto 9 stain	Thermo Fisher Scientific	Cat# S34854
Propidium Iodide	Thermo Fisher Scientific	Cat# L10316
Carboxyfluorescein diacetate succinimidyl ester (CFDA-SE)	BioLegend	Cat# 423801
Streptokinase	Millipore Sigma	Cat# S0577
Trypsin	Millipore Sigma	Cat# T4799
DNase I	Millipore Sigma	Cat# DN25
Phorbol 12-myristate 13-acetate (PMA)	Millipore Sigma	Cat# 524400
N-Formyl-Met-Leu-Phe (fMLP)	Millipore Sigma	Cat# F3506
HNP-1 (human α-defensin 1)	AnaSpec	Cat# AS-60743
FRET oligonucleotide substrate	Integrated DNA Technologies (Kavanaugh et al., 2019)	Custom synthesis
Sodium hypochlorite	ACROS Organics	Cat# 419550250
Collagen I, rat	BD Biosciences	Cat# 354236

Critical commercial assays		

iTaq Universal SYBR Green Supermix	Bio-Rad	Cat# 1725121
LDH Cytotoxicity Detection Kit	Millipore Sigma	Cat# 11644793001

Experimental models: cell lines		

Dermal fibroblasts, murine	C57BL/6J female mice, isolated according to Khan and Gasser (2016)	N/A

Experimental models: organisms/strains		

Mouse: C57BL/6J	Jackson Laboratories	RRID:IMSR_JAX:000664
Mouse: Catchup^IVM-red^: C57BL/6-*Ly6g*(tm2621(Cre-tdTomato)Arte)	Hasenberg et al., 2015	N/A

Oligonucleotides		

See Table S1		

Recombinant DNA		

pJB38; mutation generation vector, Cm^R^ / Amp^R^	Wörmann et al., 2011	N/A
pCM28; *S. aureus - E. coli* shuttle vector, Cm^R^ / Amp^R^	Pang et al., 2010	N/A
pCM29; sGFP expression vector, Cm^R^ / Amp^R^	Pang et al., 2010	N/A
pHC66; *mgrA* complementing vector (pCM28::*mgrA*), Cm^R^ / Amp^R^	Crosby et al., 2016b	N/A
pJK09; *sak* deletion vector (pJB38 *sak*::tetM), Cm^R^ / Amp^R^	This Paper	N/A
pKS80; *L. lactis* expression vector, Erm^R^	O’Brien et al., 2002	N/A
pKS80::*clfA*; vector for expression of ClfA in *L. lactis*, Erm^R^	O’Brien et al., 2002	N/A

Software and algorithms		

FIJI - ImageJ	Schindelin et al., 2012	https://imagej.net/software/fiji/
CFX Manager, v. 3.1	Bio-Rad	https://www.bio-rad.com/en-us/sku/1845000-cfx-manager-software?ID=1845000
Imaris, v. 9.5.1	Oxford Instruments	https://imaris.oxinst.com/versions/9-5
Prism, v.7	GraphPad Software	https://www.graphpad.com/scientific-software/prism

Other		

Angiogenesis μ-slide, tissue culture treated	Ibidi	Cat# 81506
8 well μ-slide, tissue culture treated	Ibidi	Cat# 80826
CFX96 Touch Real-Time PCR System	Bio-Rad	N/A
BZ-X710 microscope	Keyence	N/A
Eclipse TE2000-E microscope	Nikon	N/A
Leica TCS SP8 2-photon microscope	Leica Microsystems	N/A
InSight DeepSee laser	Spectra-Physics	N/A

## Highlights

- *S. aureus* requires the ArlRS-MgrA regulatory cascade for virulence in skin infection
- Inactivation of ArlRS or MgrA blocks *S. aureus* adhesion and abscess structuring
- The ArlRS-MgrA cascade is essential for *S. aureus* immune evasion
