# Supplementary material for: Staphylococcus aureus uses the ArlRS and MgrA cascade to regulate immune evasion during skin infection
Source: Cell Rep. Author manuscript; Available in PMC 2021 Sep 19. (PMC8450000; doi:10.1016/j.celrep.2021.109462)
Supplement: 1 [file NIHMS1728475-supplement-1.pdf]

Cell Reports, Volume *36*

Supplemental information

Staphylococcus aureus uses the ArlRS and MgrA cascade to regulate immune evasion during skin infection

Jakub M. Kwiecinski, Rachel M. Kratofil, Corey P. Parlet, Bas G.J. Surewaard, Paul Kubes, and Alexander R. Horswill

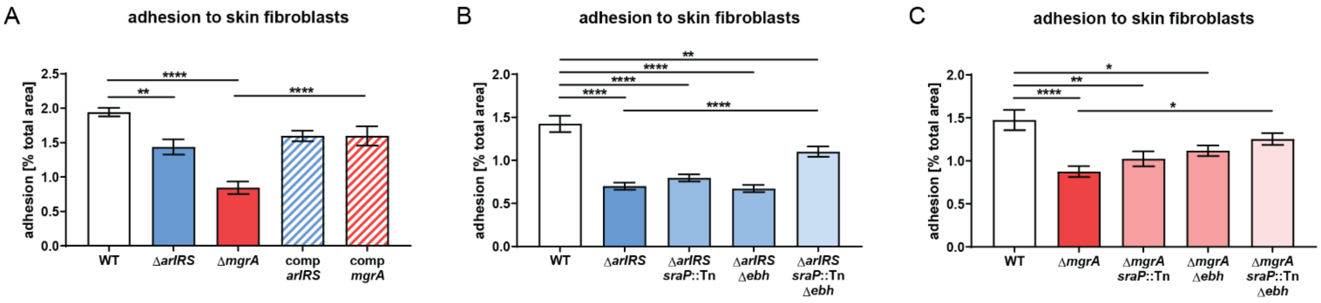

Figure S1. ArlRS and MgrA control *S. aureus* adhesion to dermal fibroblasts. Adhesion of fluorescent *S. aureus* to mouse dermal fibroblasts cultured *in vitro* was measured depending on mutations in ArlRS-MgrA regulatory system (**A**), or on presence of giant surface proteins Ebh and SraP, de-repressed in the Δ*arlRS* mutant (**B**) and the Δ*mgra* mutant (**C**). n = 10 (A); = 15 (B-C). *p* values = * <0.05, ** <0.01, **** <0.0001. All significant *p* values between the groups are marked on graphs. Related to Figure 1.

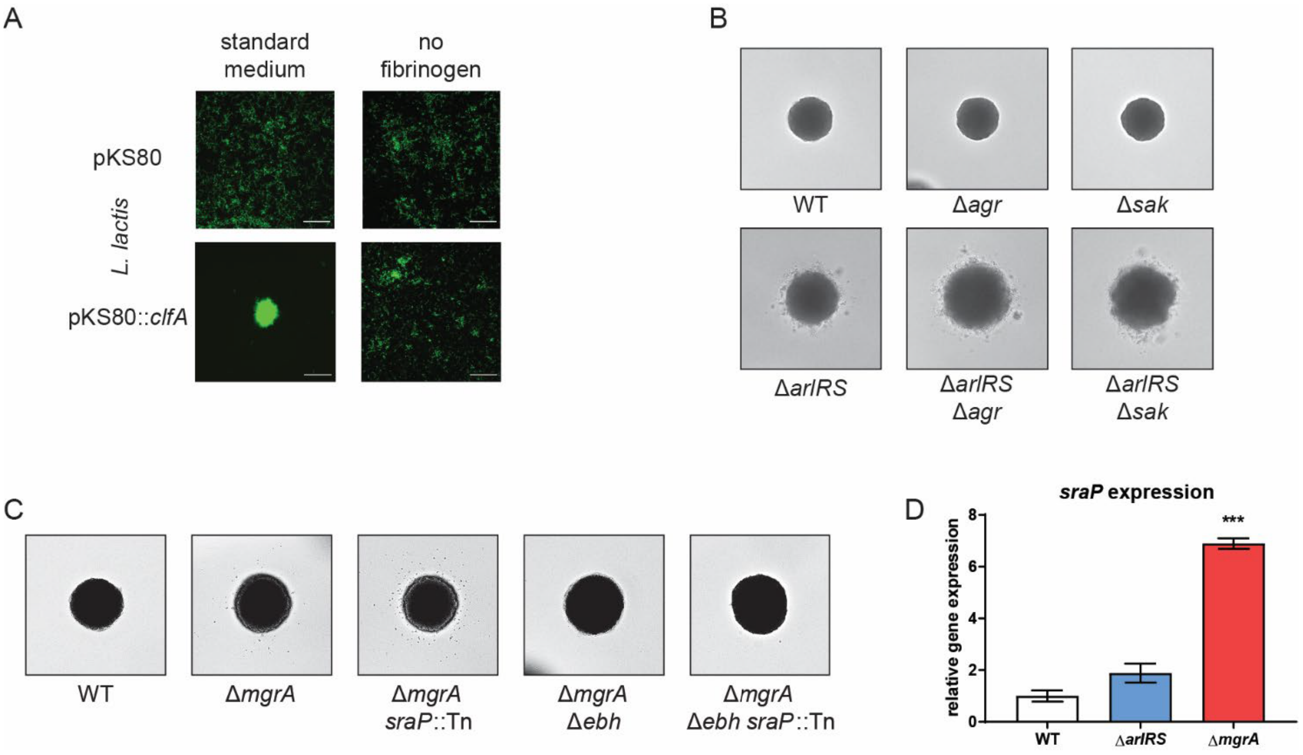

Figure S2. Binding of fibrinogen is necessary for formation of model *in vitro S. aureus* abscess communities. Model 3-dimensional staphylococcal abscess communities were formed in collagen/fibrinogen/RPMI gels. Representative images from experiments with abscess community formation by *L. lactis* heterologously expressing ClfA, stained with Syto9, are shown (**A**). Scale bar = 100 µm. Representative images from experiments determining effect of combined mutations in *agr*, *sak*, and *arlRS* in *S. aureus* this system are shown. Image size: 350×350 µm (**B**). Representative images demonstrating the role of giant surface proteins SraP and Ebh in causing the "starburst" phenotype in the Δ*mgrA* mutant strains. Image size: 350×350 µm (**C**). Expression of *sraP* in mid-exponential *S. aureus* RPMI culture was measured with qPCR and normalized to *gyrB* expression (**D**). n = 3 (**D**). *p* values = *** <0.001. Related to Figure 2.

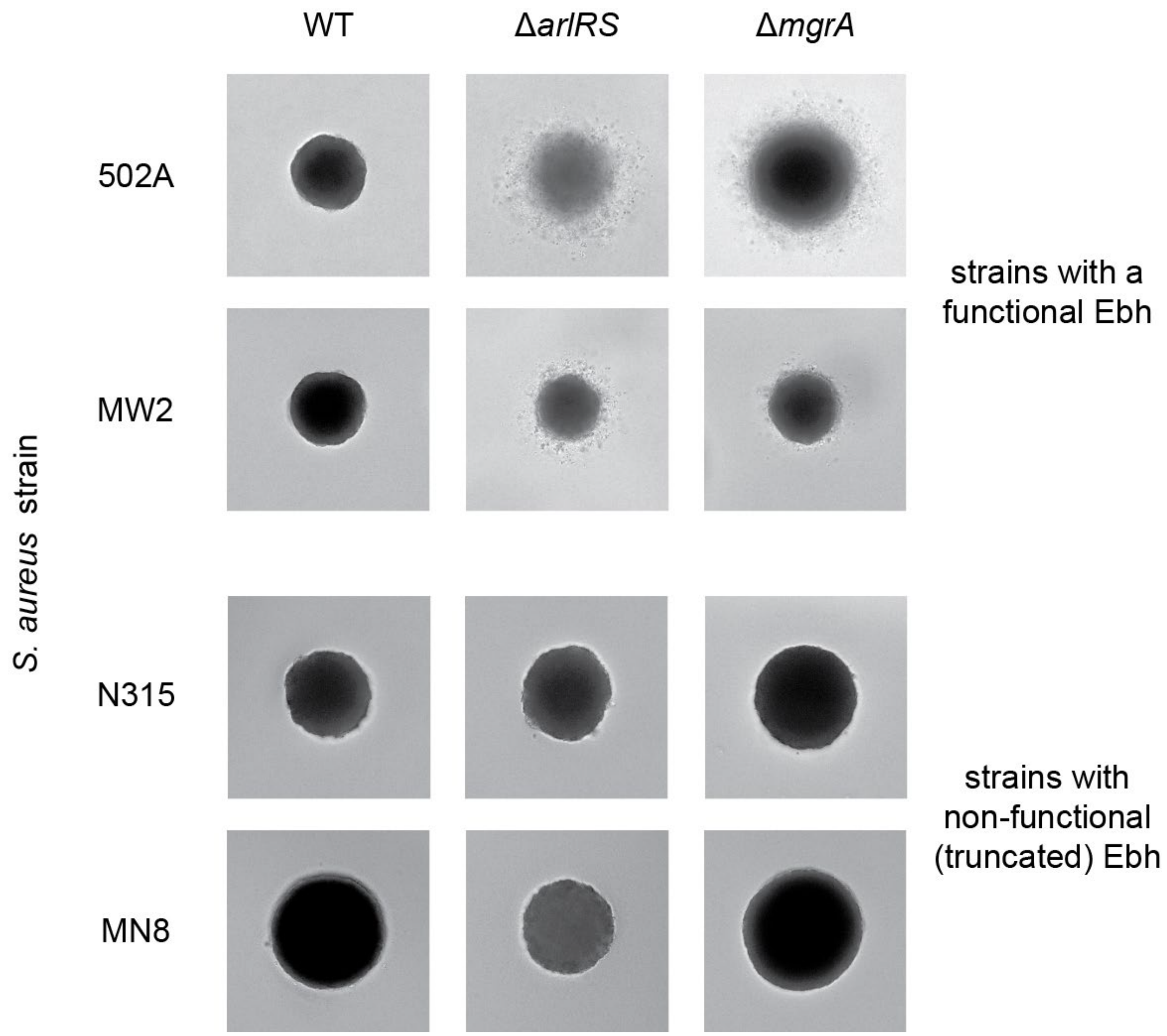

Figure S3. ArlRS regulates formation of model *in vitro S. aureus* abscess communities in various *S. aureus* strains. 3-dimensional staphylococcal abscess communities were formed from individual *S. aureus* cells belonging to different strains after culturing in collagen/fibrinogen/RPMI gels for 16h. Effect of the Δ*arlRS* and Δ*mgrA* mutations on strains possessing whole-length functional Ebh giant surface protein (strains 502A, MW2), and on strains having only truncated Ebh (N315, MN8) are shown. Representative images, image size: 350×350 µm. Related to Figure 2.

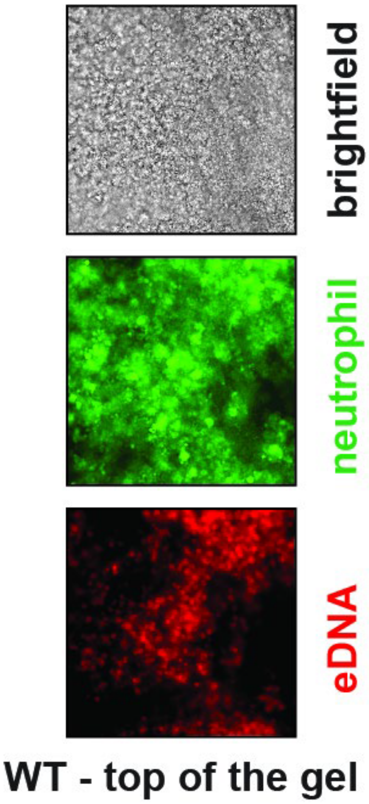

Figure S4. Neutrophils do not penetrate into gel with embedded 3-dimensional *S. aureus* abscess communities formed by WT strain. Human neutrophils (stained green with CFDA-SE) were added to 3-dimensional staphylococcal abscess communities formed by MRSA WT strain in collagen/fibrinogen/RPMI gel. After 3h co-incubation, propidium iodide was added to visualize extracellular DNA (eDNA) and dead cells. Images from the layer immediately above the gel were taken to visualize neutrophils that did not penetrate into the gel. Representative images are shown. Image size: 350×350 µm. Related to Figure 2.

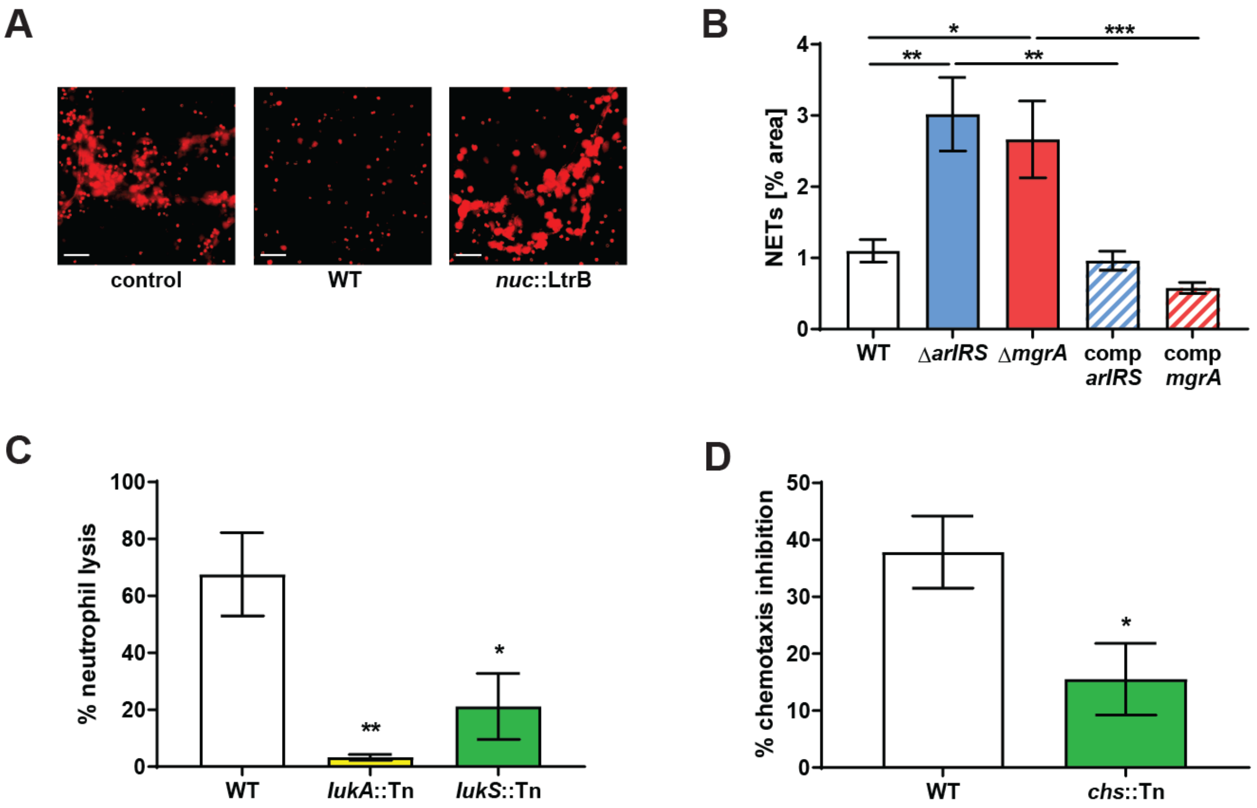

Figure S5. Innate immune evasion mechanisms employed by *S. aureus* are attributable to specific virulence factors. The ability of *S. aureus* culture supernatants to digest neutrophil extracellular traps, visualized with propidium iodide (**A**), to kill human neutrophils (**C**) and to block neutrophil chemotaxis (**D**) was measured in WT strain and in mutants lacking selected virulence factors. Additionally, degradation of neutrophil extracellular traps by culture supernatants of *S. aureus* MRSA WT and its Δ*arlRS* and Δ*mgrA* mutants was quantified (**B**). Representative images are shown. Scale bar = 100 µm. Data shown as mean ± SEM. n = 8 (**B**); = 4 (**C**); = 5 (**D**). For **A**, representative images are shown, scale bar = 100 µm. *p* values = * <0.05, ** <0.01, *** <0.001. Related to Figure 3.

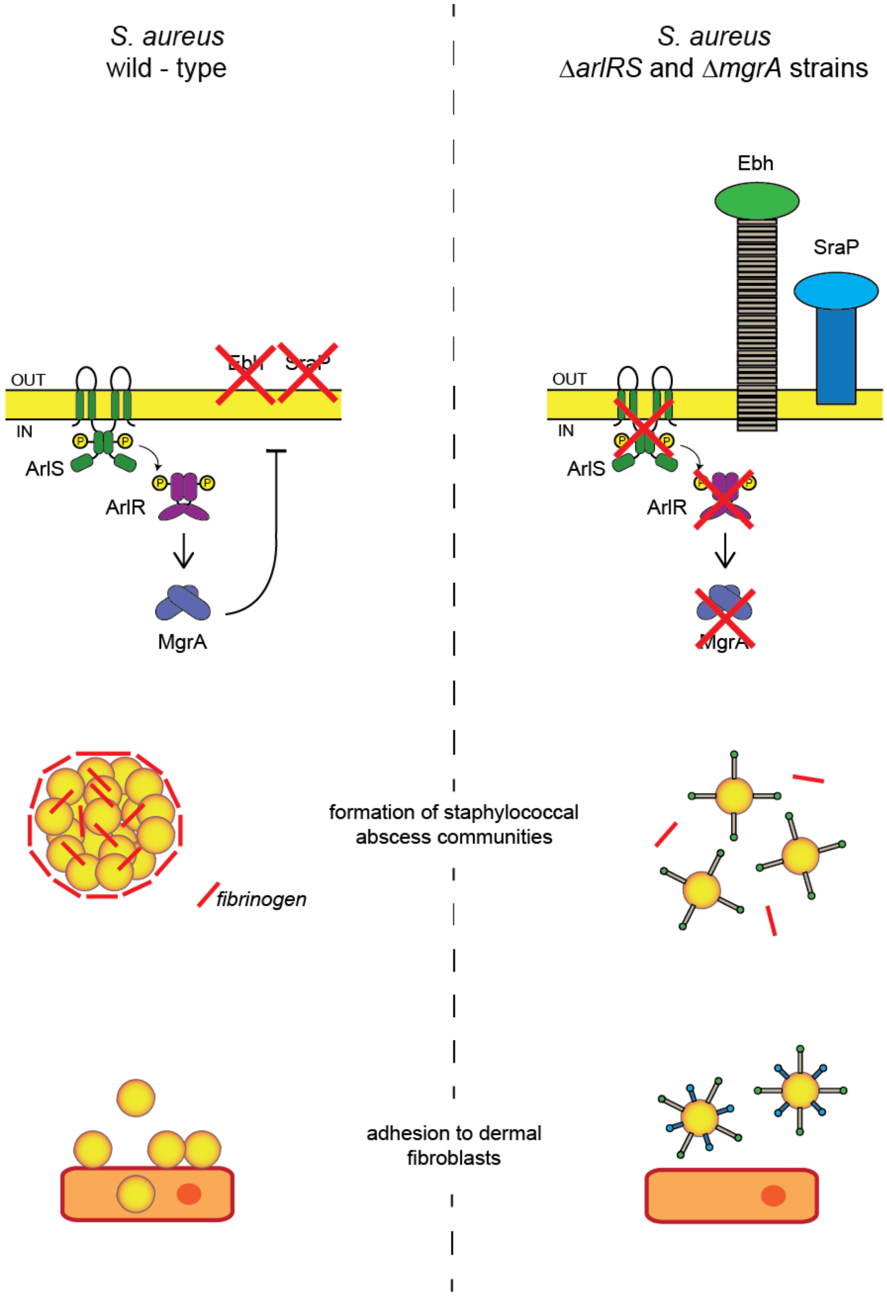

Figure S6. A model of ArlRS and MgrA affecting spatial organization of *S. aureus* during skin infection. With a functional ArlRS-MgrA cascade, the large surface proteins with anti-adhesive properties (Ebh, SraP) are repressed during skin infection. This allows *S. aureus* to bind fibrinogen and to organize itself into staphylococcal abscess communities: tight 3-dimensional structures encased in fibrinogen. It also allows *S. aureus* to adhere to host cells in the skin. However, when the ArlRS or MgrA elements of the regulatory cascade are absent, the large proteins Ebh and SraP become de-repressed and appear on staphylococcal surface. By interfering with fibrinogen binding, Ebh prevent formation of a staphylococcal abscess community. The joint inhibitory activity of Ebh and SraP also prevents binding of *S. aureus* to skin fibroblast. Lack of proper spatial organization leaves *S. aureus* in the skin as individual, non-attached cells, susceptible to phagocytosis by host immune cells. Related to Figure 7.

Supplementary Table 1. Oligonucleotides used in this study. Related to STAR methods.

Code	Name	Sequence
		**gene deletion**
JK41	sak_A2	gaggccctttcgtcttcaagaattcggtgatgtggctgtatttaccaaag
JK42	sak_B2	ctattttgttttaggtaccgagcatggcgcttcctcc
JK43	sak_C2	cgccatgctcggtacctaaaacaaaatagttgtttattatagaaag
JK44	sak_D2	agagcttgcatgcctgcaggtcgacgcactggtgaattcttttc
		**qPCR**
JK69	chs_qPCR_F1	caggaatcagtacacaccatc
JK70	chs_qPCR_R1	gcgttgtaggaagaccactatt
JK71	lukA_qPCR_F2	agctcaggtggtaaattcgattc
JK72	lukA_qPCR_R2	gaccagtgtacatgccagttatt
JK73	lukB_qPCR_F2	ggacatgaccatacgagacaat
JK74	lukB_qPCR_R2	aacccttcagacacagttacag
JK75	scin_qPCR_F3	aaatctatacttgcgggaactt
JK76	scin_qPCR_R3	aagcttgtgctagcttgtg
HC314	sraP_1738_for	actgtaggcaatcaaaccataga
HC315	sraP_1851_rev	ccgcttggtaatcctgtaact
HC505	nuc83_for	cgaaagggcaatacgcaaag
HC506	nuc159_rev	tgcatttgctgagctacttaga
HC579	lukS_up	ctgcaacattgtcgttaggaataa
HC580	lukS_down	ctcagcgccatcaccaata
HC581	lukF_up	ggcttatcaggtggaggtaatg
HC582	lukF_down	gcttcaacatcccaaccaattt
4252024X	SA-ebh7908F	tgcgaagaagcgtgaagcagaaac
4252023X	SA-ebh8091R	ttgttgcactgcttgctctaaggc
41995031X	SA-gyrBFor	aacggacgtggtatcccagttgat
41995030X	SA-gyrBRev	ccgccaaatttaccaccagcatgt
